# COVID-19 Disease and Economic Burden to Healthcare Systems in Adults in Six Latin American Countries Before Nationwide Vaccination Program: Ministry of Health Database Assessment and Literature Review

**DOI:** 10.3390/ijerph22050669

**Published:** 2025-04-24

**Authors:** Natalia Espinola, Cecilia I. Loudet, Rosario Luxardo, Carolina Moreno, Moe H. Kyaw, Julia Spinardi, Carlos Fernando Mendoza, Carolina M. Carballo, Ana Carolina Dantas, Maria Gabriela Abalos, Jamile Ballivian, Emiliano Navarro, Ariel Bardach

**Affiliations:** 1 Department of Health Technology Assessment and Health Economics, Institute for Clinical Effectiveness and Health Policy (IECS), Buenos Aires City C1414, Argentina; cloudet@med.unlp.edu.ar (C.I.L.); rosario.luxardo@gmail.com (R.L.); carolina.morenoudea@gmail.com (C.M.); jballivian@iecs.org.ar (J.B.); licnavarro1985@gmail.com (E.N.); abardach@iecs.org.ar (A.B.); 2 Vaccine, Medical, Pfizer Inc., New York, NY 10001, USA; moe.kyaw@pfizer.com; 3 Vaccine, Medical, Emerging Markets, Pfizer Inc., Itapevi 06696-000, Brazil; julia.spinardi@pfizer.com; 4 Vaccine HTA, Value and Evidence, Pfizer Inc., Mexico City 05120, Mexico; carlosfernando.mendoza@pfizer.com (C.F.M.); mariagabriela.abalos@pfizer.com (M.G.A.); 5 Vaccines, Medical, Pfizer Inc., Buenos Aires City C1437, Argentina; carolina.carballo@pfizer.com (C.M.C.); ana.dantas@pfizer.com (A.C.D.); 6 Centro de Investigaciones Epidemiológicas y Salud Pública (CIESP-IECS), Consejo Nacional de Investigaciones Científicas y Técnicas (CONICET), Buenos Aires City C1425FQD, Argentina

**Keywords:** adults, COVID-19, disease burden, economic burden, Latin American

## Abstract

The COVID-19 pandemic imposed a substantial burden on healthcare systems worldwide, yet reliable data on COVID-19 morbidity, mortality, and healthcare costs in Latin America remain limited. This study explored the disease and economic burden of COVID-19 in Argentina, Brazil, Chile, Colombia, Mexico, and Peru during the pre-vaccination period. Using national databases and a systematic review of the literature, we analyzed data on adults aged 18 and older, reporting cases, death rates, years of life lost, excess mortality, and direct medical costs. Before vaccination programs began, the average COVID-19 incidence rate was 6741 per 100,000 adults. Of these, 91% were mild cases, 7% moderate/severe, and 2% critical. Among 2,201,816 hospitalizations, 27.8% required intensive care, and 17.5% required mechanical ventilation. Excess mortality ranged from 76 to 557 per 100,000, and years of life lost spanned 241,089 to 3,312,346. Direct medical costs ranged from USD 258 million to USD 10,437 million, representing 2–5% of national health expenditures. The findings highlight significant variability across countries and provide crucial insights to help policymakers to make informed decisions and allocate resources effectively to improve national strategies around surveillance, preventive and treatment strategies to control the spread of COVID-19 disease in the future.

## 1. Introduction

The COVID-19 pandemic placed substantial strain on healthcare systems worldwide. Latin America has been particularly hard-hit, with several countries reporting high cases and death tolls [1,2]. The region has accounted for approximately 15% of global cases and 28% of global deaths [3]. Beyond the known health risks to the population, the COVID-19 pandemic has had significant economic repercussions for the health system and society in a region already facing structural difficulties. This impact on the region has been significant, particularly for vulnerable groups, such as low-income families, racial minorities, older adults, those with high job loss and business closures, and among young parents and those with lower incomes [2,4,5,6].

The roll-out of COVID-19 vaccination has been a significant public health effort worldwide. Governments, healthcare organizations, and pharmaceutical companies have collaborated to develop, manufacture, and distribute vaccines at an unprecedented pace [7]. The roll-out of COVID-19 vaccines has been a complex process, involving prioritizing high-risk populations, ensuring equitable access to vaccines, and addressing vaccine hesitancy [8]. Despite these challenges, many countries have made significant progress in vaccinating their populations [9]. The success of the vaccine roll-out was crucial for controlling the spread of COVID-19 [10]. How to sustain the vaccination program, considering its impact on the community in the post-pandemic scenario, is still a gap to be understood. Pandemics and other health crises vary in their intensities and impacts, each requiring a distinct set of preventive policies. Selecting short-term policies to minimize damage demands careful data analysis, but in the early stages of a crisis, the available information is often limited, imperfect, and noisy. This uncertainty makes it difficult for policymakers to make optimal decisions in real time. Given that pandemic preparedness intersects with public health, national security, and economic competition, understanding the key technology and policy trends of major global stakeholders is essential [11,12]. In this context, understanding the COVID-19 disease and economic burden to healthcare systems before vaccination is crucial. Our main objective was to estimate the burden of COVID-19 in terms of incidence, hospitalization, and mortality, as well as its cost to the healthcare system, in the adult population before vaccination in six countries in the Latin American region.

## 2. Materials and Methods

To estimate the burden of disease and perform cost analyses in adults from six Latin American countries (Argentina, Brazil, Chile, Colombia, Mexico, and Peru) [13], we performed a comprehensive review of official statistics databases (such as surveillance systems, national records of the cause of death, and hospitalization when available). We considered the period before COVID-19 vaccination (from inception until 1 June 2021 (Argentina, Brazil, Colombia, Mexico, Peru) or 1 February (Chile)) and information that was publicly available. This timeframe was determined considering that there was no nationwide vaccination program in place, meaning that no more than 30% of the population had received a single dose of the vaccine and the first two waves of the pandemic were included. When required, we contacted the Ministries of Health officers to access COVID-19 databases to estimate the outcomes of interest for this study. The World Health Organization database was also used for this analysis. Supplementary Material Section S1 provides information on the sources (Table S1), criteria related to the official databases, and flowcharts (Figures S1–S6) used to determine the population included in the final analysis for each country. We supplemented the data through a systematic literature review when official databases needed more information.

### 2.1. Inclusion and Exclusion Criteria

This analysis only included studies that provided data from the adult general population (≥18 years) diagnosed with COVID-19 in Argentina, Brazil, Chile, Colombia, Mexico, and Peru in the pre-vaccination period. We searched the data and stratified the population by sex (female-male) and age (18–49, 50–64, and ≥65 years). Also, we searched data on individuals with certain comorbidities (hypertension, diabetes, obesity, chronic kidney disease, asthma, chronic obstructive pulmonary disease, cardiovascular disease, and other forms of immunosuppression). We included surveillance studies, prevalence cohorts, ministerial reports, WHO reports, budget impact analyses, and economic or direct cost evaluations using a microeconomic approach. Randomized clinical trials were excluded from this study. We also excluded reports from Chile after 1 February 2021 and reports from Argentina, Brazil, Colombia, Mexico, and Peru after 1 June 2021, to comply with the established pre-vaccination period.

### 2.2. Design of Studies Included

We included studies with any epidemiological or economic design (whose full text was Spanish, English, or Portuguese). Epidemiologically relevant surveillance reports for the selected calendar period were also assessed. We also included observational studies, such as cohort, case–control, case series, economic evaluations, and cost or budget impact studies.

Study selection was performed using COVIDENCE, a web-based platform designed for the systematic review process [14]. The authors of the articles were contacted when necessary to obtain missing or supplementary information. Previously piloted in five studies, a predesigned general data extraction form was used (available on demand). Disagreements were resolved by consensus of the entire team.

### 2.3. Risk of Bias

The risk of bias in observational studies was assessed using a checklist developed by the US National Heart, Lung, and Blood Institute, which classifies studies as having a high risk of bias (poor), uncertain (fair), and low risk of bias (good) [15]. For the assessment of cohort and cross-sectional studies, the tool comprises 14 items, while 9 items apply to case series studies. The assessments were independently performed by peer reviewers from the research team. These tools are well suited to the observational studies predominantly represented in our dataset. Any discrepancies were resolved through team consensus. Several potential limitations of the literature review include incomplete data in published studies and MOH reports, heterogeneity and quality of available data, and publication bias due to the inclusion of only one geographical region in the review. Supplementary Material Section S2 shows the literature search and flowchart (Figure S7) of the included studies.

### 2.4. Epidemiological and Cost Outcomes

The primary health outcomes analyzed were cases defined by the National Health Ministries, deaths, and types of hospitalizations by severity stratified by age group and sex. According to WHO Clinical Practice Guides for the Management of COVID-19 published in 2021, COVID-19 was classified into different categories: mild, moderate, severe, and critical [16]. Firstly, mild COVID-19 is identified in cases requiring symptomatic treatment and not presenting viral pneumonia or hypoxia. On the other hand, cases with non-severe pneumonia are considered moderate COVID-19 cases, and the designation of severe COVID-19 is reserved for patients with pneumonia of considerable severity. Finally, COVID-19 is defined as critical to those patients who present with Acute Respiratory Difficulty Syndrome (ARDS). However, in the analysis of the databases, it was found that the countries did not use this classification for COVID-19 cases but, rather, classified them according to the need and type of hospitalization. Therefore, to homogenize the information and use the WHO categorization, the aforementioned definitions were used. However, the definitions of moderate and severe are diffuse and very difficult to categorize according to the type of hospitalization, so we had to unify these two categories into one called moderate and severe.

We also estimated the years of life lost (YLLs), potential years of life lost, disability-adjusted life years (DALYs), and excess mortality (EM). YLLs are defined as the difference between an individual’s age at death and their life expectancy, indicating how many years that person could have lived if they had not died prematurely [4,17]. To identify these outcomes, we conducted a systematic review and prioritized studies with the widest analysis period (aligned with our study’s timeframe) and reported both total and sex-disaggregated YLLs. We also reported the overall YLLs and age-standardized YLLs (further details are available in Supplementary Material Section S3). The WHO database also estimated the excess mortality attributed to COVID-19 (Supplementary Material Section S4). The WHO defines excess mortality due to COVID-19 as the difference between the number of observed deaths from all causes during a given period and the number of expected deaths from the same period, based on historical data from recent years [18].

The economic burden per patient associated with COVID-19 was calculated using a cost-of-illness analysis through the micro-costing approach [19]. The perspective of the analysis was the healthcare system. This method consisted of identifying health resources, rates of use, and quantities, then assigning unit costs to each resource.

For the identification of healthcare resources and their use rates, we relied on the WHO guide on managing COVID-19 [20], supplemented with the systematic review, the opinion of local experts with extensive experience in managing COVID-19 patients in ICU settings, which can provide valuable insights into rates of use in real life. Only direct medical costs were considered in this analysis, including drug acquisition and administration costs, disease monitoring (laboratory tests and imaging), hospitalization (General Ward, ICU, and ICU with mechanical ventilation), and medical consultation. We assumed that the rates of use and quantities of most healthcare resources (consultations, laboratory examinations, diagnostic tests, and medications) do not differ between countries, since they are based on good clinical practice, except in the case of length of hospital stay, where we differentiate between countries using information obtained from the literature review and indirect estimates through the benefit transfer method [21,22,23]. With this information, it was possible to estimate the duration of hospitalization in general wards, ICU without mechanical ventilation, and ICU with mechanical ventilation, according to the level of severity. Supplementary Material Section S5 details the length of stay in days (Table S12) and the rate of use of healthcare resources based on case severity (Table S13).

Unit healthcare cost databases were used for each country [24,25,26,27], except for Mexico and Peru, where costs had to be estimated indirectly due to the lack of country-specific information. Drug costs were collected from different sources, depending on the country [15,16,17,28,29,30]. In the case of Mexico, the drug costs were calculated by indirect estimation using the benefit transfer method [8,21]. Details of the unit cost approach and information by severity and country are shown in Supplementary Material Section S6.

The results are presented as the cost per patient associated with COVID-19, according to the severity of the disease and the total cost faced by the country (multiplying the cost per patient by the number of cases in the country). The costs were updated for inflation using the Consumer’s Price Index obtained from the official pages of each country to June 2023 [31,32,33,34,35]. Costs are reported, based on each country’s nominal exchange rate, in USD for 2023 [36,37,38,39]: Argentina: ARS/USD 350.00/1; Brazil: BRL/USD 4.90/1; Chile: CLP/USD 855.66/1; Colombia: COP/USD 4066.89/1; Mexico: MXN/USD $6.98/1; Perú: PEN/USD 3.69/1.

In addition, we explored the disease and economic burden of other preventable diseases (influenza and Invasive Pneumococcal) before vaccine introduction in the National Immunization Program (NIP) for reference. It is essential to highlight that disease comparisons are inadequate due to differences in burden, epidemiological settings, age prevalence, surveillance system, and agent characteristics, among others. We retrieved data from 2001 to 2017 from ministerial reports from every country and the Surveillance Network System of Agents Responsible for Bacterial Pneumonia and Meningitis (SIREVA). This period was selected based on the vaccination timelines for each country. A complementary literature search was conducted using the PubMed and Lilacs databases to estimate the costs associated with the selected diseases (see Supplementary Material Section S7). Studies of the economic burden or direct medical costs were identified for each vaccine-preventable disease in Argentina, Brazil, Chile, Colombia, and Peru.

Regarding other vaccine-preventable diseases such as influenza and S. pneumoniae pneumonia, we also assessed health outcomes in adults aged 18 years and above before introducing vaccines for these conditions in the national immunization schedule. The search methodology is described in Supplementary Material Section S8.

### 2.5. Statistical Analysis

The overall incidence rates for cases, deaths, and hospitalizations (per 100,000 persons) were extracted from the Ministry of Health (MOH) database for each country (see Table S1 in Supplementary Material). Regarding YLL, age-standardized estimates for people over 20 years of age were derived from data from the Ministry of Health (MOH) database and information extracted from the included studies in the systematic review (Supplementary Material Section S3). Excess mortality per 100,000 persons, encompassing all age groups in the study population, was also assessed using data from the studies included in the systematic review. The population information for estimating excess mortality rates was sourced from the World Health Organization (WHO) database. Descriptive statistics, including frequency and percentage, were employed. A subgroup analysis was performed based on age (18–49, 50–64, and ≥65 years) and sex. Data were analyzed with R software (4.2.1). Table S18 Supplemental Material Section S9 shows the data population by country.

## 3. Results

**Figure 1:**
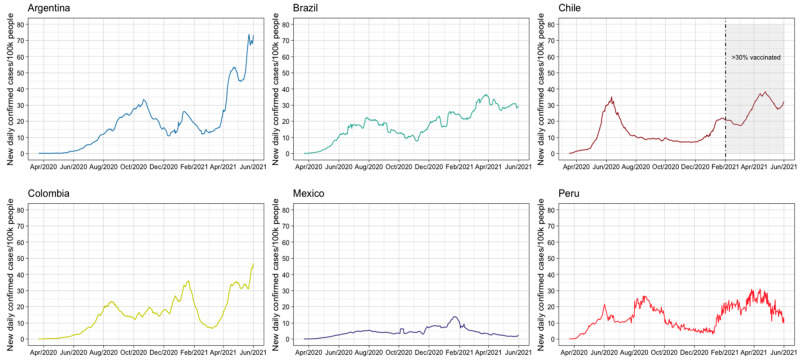
Daily new confirmed COVID-19 cases per 100,000 persons. Notes: elaborated from open-source data from Our World in Data [27]. and normalized to 100,000 persons by country.

The number of COVID-19 cases diagnosed by the health system ranged between 700,000 (Chile) and 11,000,000 (Brazil). The highest incidence rate of COVID-19 cases was observed in patients under 65 years of age in all countries (Table 1). According to the WHO definition of severity, approximately 90% of the cases were mild, 7% were moderate and severe, and 3% were critical. Generally, males had a higher incidence rate than females in the selected countries (nearly 7 percentage points difference), except for Colombia. Figure 1 presents the daily confirmed COVID-19 cases per 100,000 individuals in the six countries, encompassing both waves. Chile captured the early second wave, while Argentina highlighted its peak. While vulnerable groups are mentioned to provide context, our primary measures are limited to age, sex, and clinical severity due to data constraints.

### 3.1. Hospitalizations

Table 2 summarizes the hospitalized COVID-19 cases (sex and age), critical care admission, and hospitalized cases with mechanical ventilation by country. Overall, 9.2% of cases required hospitalization, with a heterogeneous distribution across countries. The majority of hospitalized cases were male (56%) and over 65 years old (39%). A similar distribution was observed in the critical care setting in all countries except Mexico and Peru, where the largest proportion was represented by the age group of 50–64 years. Sex- and age-stratified patients under critical care are summarized in Table S19A in the Supplementary Material. Less than 20% of the patients required mechanical ventilation. Sex- and age-stratified patients under mechanical ventilation are summarized in Table S19B of the Supplementary Material.

Data on COVID-19 individuals with certain comorbidities are summarized in Table S20 of the Supplementary Material. The majority of the analyzed cohorts had diabetes (7.5 to 23.2%) or hypertension (14.4 to 40.8%) as associated comorbidities in all countries. Brazil reported the largest proportion of COVID-19 cases with diabetes (20.3%), whereas Colombia reported the largest proportion of cases with hypertension (40.8%).

### 3.2. Mortality

A total of 1,144,544 people over 18 years of age died of COVID-19 during the selected period, representing 5% of the overall cases. Even though the majority of deaths were male (59%), when stratified by age and sex, a larger proportion of females over 65 years of age died than males of the same age group. Table 3 summarizes death counts and percentages by country, sex, and age. The case fatality rate during the pre-vaccination period varied between countries from 2.5% (Argentina and Chile) to 10% (Mexico and Peru) (Table 3). As shown in Figure S8 in the Supplemental Material, the monthly CFR trajectories revealed elevated CFR rates for Mexico and Peru, confirming the global CFR data.

### 3.3. General Characteristics and Risk of Bias of the Included Studies

The systematic review included 20 studies conducted across multiple Latin American and multinational settings, with analysis periods ranging from 4 to 18 months. Data sources varied, including national health ministries, mortality databases, hospital records, and civil registries. All studies employed observational designs, with most assessing excess mortality and P-score (nine studies), followed by years of life lost (YLLs/PYLLs) (five studies), hospital mortality (four studies), incidence rates (one study), and case fatality rates (one study). The risk of bias was generally rated as fair (14 studies) or good (6 studies), with no studies classified as poor. These findings highlight the variability in study design and data quality, underscoring the importance of methodological considerations in interpreting the pandemic’s impact across different health metrics and regions (see Table S2 Supplementary Material).

### 3.4. Years of Life Lost and Excess Mortality

We identified and prioritized five studies that estimated the burden of COVID-19 and used the DALYs and/or YLLs as metrics (Table S2). The overall analyses of YLLs primarily rely on reports from multiple countries based on ministerial databases, encompassing different analysis periods throughout 2020, ranging from eight months to one year (Table S3). The Table 4 shows the YLL and DALYs per 100,000 persons by country. There was high variability in the estimation of YLL, ranging from 241,089 in Chile to 3,312,346 in Brazil. In addition, Peru and Mexico had the highest DALYs per 100,000 people, ranging from 2549.5 to 3510.7.

**Figure 2:**
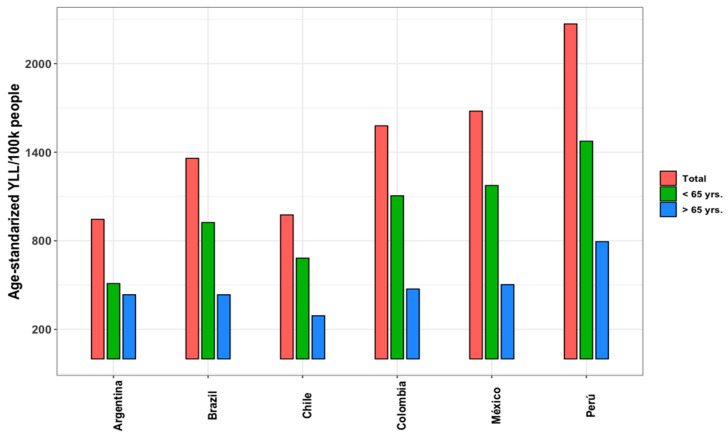
Total age-standardized years of life lost rates from COVID-19 by country and age group *. Notes: using data from Salinas-Escudero, 98.6% of total YLL rates were employed to estimate age-standardized YLL rates for individuals aged 20 years and above [29]. Source: All data were retrieved from the studies shown in Table S2. * Period under analysis pre-vaccination.

Based on the data provided in these reports, we estimated age-standardized YLL for the six Latin American countries stratified by age group (Figure 2). For more details, see Tables S4–S9 in the Supplemental Material. The age-standardized YLL per 100,000 persons ranged from 945 in Argentina to 2268 in Peru. Younger patients (<65 years) contributed more to YLL rates than adults aged >65 years.

**Figure 3:**
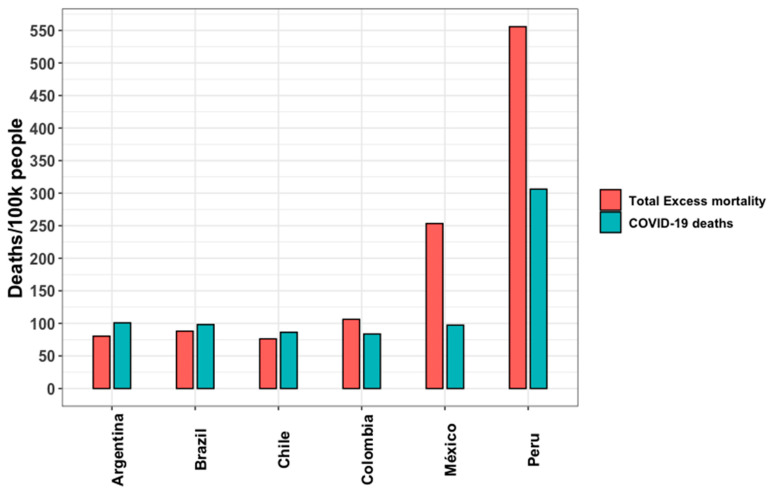
All age ranges of total excess mortality and COVID-19 deaths per 100,000 persons in six Latin American countries in 2020. Source: author’s elaboration based on the literature review.

Regarding excess mortality, we prioritized the five papers identified through a systematic review. The characteristics of the included studies are shown in Table S2. The analysis period encompassed almost all cases in 2020; however, significant variations in excess mortality were observed, ranging from a p-score of 10.6 to 109.5 (Table S10). The total excess mortality per 100,000 people ranged from 76 to 557 in Argentina and Peru, respectively (Figure 3). In Argentina, Brazil, and Chile, COVID-19 deaths have exceeded excess mortality, while in Colombia, Mexico, and Peru, excess mortality rates have surpassed those of COVID-19 deaths. Regarding age, excess mortality data were accessible only for Mexico and Brazil (Supplementary Table S11), revealing that the overall excess mortality and the proportion of excess deaths attributable to COVID-19 were greater among males than females.

### 3.5. Economic Burden of COVID-19

Table 5 details the direct medical costs related to COVID-19, classifying them according to the cost components and disease severity in specific countries. The costs per case of mild COVID-19 ranged from USD 26.6 in Peru to USD 68.9 in Argentina. In mild cases, diagnosis and laboratory tests represent the resource with the greatest share of the total cost in Argentina, Brazil, Mexico, and Peru. By contrast, in Chile and Colombia, medical consultations are the largest component of the cost per mild case.

The costs for moderate and severe cases vary between USD 1059.4 in Brazil and USD 2951.3 in Chile, with hospitalization being the resource with the greatest weight in the cost for this type of case in all the countries analyzed.

On the other hand, the costs per critical case ranged between USD 7147.2 in Colombia and USD 30,040.9 in Mexico. In Argentina, Brazil, and Mexico, the resource with the greatest impact in these cases is related to drug spending, whereas in Chile, Colombia, and Peru, the most significant component is associated with hospitalization.

Therefore, the total cost associated with COVID-19 ranged from USD 250 million in Peru to USD 10 billion in Brazil. This represents 2% of the total health spending per country, except for Brazil, where it represents 5% (see Table 6). Critical cases represent the largest proportion of the total costs due to COVID-19. However, in the case of Peru, moderate and severe cases contribute more to the total cost, given that the proportion of critical cases in Peru (0.6% of total COVID-19 cases) is relatively lower than in other countries.

### 3.6. Other Vaccine-Preventable Diseases

Data from pre-vaccinal cases of pneumonia and influenza in the selected countries presented substantial heterogeneity, probably given the different onset of vaccination programs and dates of the data. Table S16 in the Supplementary Material summarizes the average death counts of pneumonia and influenza. Argentina, along with Brazil, experienced the highest number of deaths compared to the other countries.

The costs per case of hospitalization due to pneumococcus found in studies vary between USD 800 and USD 1900 in Argentina [42], Brazil [43], and Colombia [44,45,46]. On the other hand, three studies from Argentina [47,48] and Peru [49] were identified for the costs per case of hospitalization due to influenza that reported costs between 20USD and USD 400. Further details on the results regarding other vaccine-preventable diseases are presented in Supplementary Material Section S8.

## 4. Discussion

In this study, we provided a comprehensive overview of the COVID-19 burden in six countries of the Latin American region before implementation of the nationwide vaccination program. These countries lead the economic income of the region and represent approximately 80% of the Latin American population. Our main finding was a substantial disease burden across all six countries, with variations in all the outcomes considered.

We observed a high degree of variability in disease burden (incidence, hospitalization, death, excess mortality, and years of life lost), and direct medical costs could be attributed to the diverse strategies employed by each country during the peak of the pandemic. These strategies include factors, such as mandatory testing in hospital settings, delays in test results, discrepancies in case reporting, and variations in case definitions, particularly before the availability of vaccines [50]. In addition, the availability of healthcare resources and treatment is also likely to impact these estimates. Crossing the six countries, most showed mild disease rates close to 90%, whereas severe disease rates were less than 4%. It is crucial to note that these proportions can be influenced by surveillance methods, therapeutic use, interventions, regional demographics, vaccination rates, and emerging variants. In national databases, assigning severity categories and determining hospital or ICU admissions could depend on various factors, including clinical judgment and local and national policies, rather than prognosis prediction models [51]. In four of the six countries analyzed, the hospitalization rate was equal to or less than 7%. This pattern may seem to yield lower results compared to findings reported in the literature from high-income regions [52,53]. However, these data are not entirely comparable, as our analysis is derived directly from national databases involving all diagnosed adult cases and the corresponding percentage of hospitalizations. In contrast, cohort reports only include hospitalizations and their associated risk factors, neglecting to consider the broader context of population data for cases.

Regardless of hospitalization policies, individuals aged 65 and older consistently constitute the demographic with the highest hospitalization and ICU admission rates in several countries (including Argentina, Brazil, Colombia, and Mexico). This elevated risk aligns with their age and comorbidities and continues to be a prevalent concern. Data from the US Centers for Disease Control and Prevention (CDC) covering January to August 2023 indicated that a significant 63% of COVID-19-related hospitalizations in 13 US states involved individuals aged 65 or older. The majority of these patients had at least two underlying medical conditions, and over three-quarters had not received a recommended dose of the bivalent COVID-19 vaccine, as advised by the CDC’s Advisory Committee on Immunization Practices in the fall of 2022 for enhanced protection against COVID-19 [54].

Another remarkable result was that, in most cases, the percentage of patients needing mechanical ventilation was nearly half of those requiring critical care. However, this relationship was more pronounced in Peru and Mexico, with 80% for Peru and almost twice as high for Mexico. One plausible explanation is that hospitals had to provide mechanical ventilation outside traditional ICUs due to high demand. This was evident early in the pandemic, before widespread vaccination. In a Mexican cohort study, 45% of non-survivors with critical illness requiring ICU admission did not receive Invasive Mechanical Ventilation (IMV) or ICU care due to limited ICU beds. A similar scenario was reported for the entire Metropolitan area of Mexico City, indicating a shortage of ICU beds and hospital overcrowding, leading to delayed ICU admissions [55].

Regarding mortality indicators, Peru experienced the most severe situation, a fact corroborated by other authors [56]. This situation likely stemmed from the health system having limited capacity and fewer resources to cope with the unfolding pandemic, as reported in other regions as well [57,58]. However, it should be noted that the Peruvian government, at the end of the first wave, changed the method of recording deaths, almost doubling the registered deaths with the new, more sensitive method, which may have influenced these significant differences [59]. In terms of age-specific distribution, the demographic group experiencing a higher mortality rate was those aged 65 and above, as consistently reported worldwide. Interestingly, when comparing both sexes across all countries, females in the age group of 65 and above displayed a higher mortality rate than males—a reversal of the trend observed in the majority of publications [52].

The similarity in values between YLLs and DALYs was primarily due to the fact that the main component of DALYs was mortality, while the disability weight associated with the acute phase of the disease was lower [60]. On the other hand, in countries where COVID-19 deaths exceeded excess mortality, one possible reason may be the decrease in mortality from different causes (such as trauma, seasonal viruses and cardiovascular problems) due to the mobility restrictions imposed and the isolation measures adopted by policymakers [61], while in countries where the opposite is the case, one possible explanation could be the tensions in the health system that limit access to conditions such as cancer and heart disease [61,62]. Underreporting, especially in LMICs, may widen this gap [59].

In economic terms, this study reveals that before vaccination implementation, total direct medical costs related to COVID-19 represented approximately 2% of total health spending in most cases, except Brazil (5%). A study on the economic burden of COVID-19 in Iran was found [49], which concluded that the total cost in 2020 was equivalent to approximately 7% of health spending. On the other hand, an analysis carried out in Saudi Arabia between March 2020 and January 2021, in a sample of 5286 patients [63], revealed that the medical costs of ICU hospitalization per patient averaged USD 4580. Comparatively, in our study, critically ill patients requiring ICU care had a total hospitalization cost of between USD 3807 and USD 8612.

In our study, the direct medical costs of COVID-19 before vaccination ranged from USD 3807 to USD 8612 per ICU patient across six Latin American countries, representing about 2% of total health spending in most cases, except Brazil at 5%. Comparatively, national health expenditures in 2020 as a percentage of GDP were as follows: Argentina (9.5%), Brazil (9.6%), Chile (9.1%), Colombia (7.7%), Mexico (5.4%), and Peru (5.2%) [1]. With GDPs in 2020 ranging from USD 191 billion (Peru) to USD 1.44 trillion (Brazil) [2], COVID-19 costs imposed a notable burden, particularly in Brazil, where 5% of a 9.6% health-to-GDP ratio highlights significant strain. This contextualization underscores the relative economic impact on healthcare budgets, supporting the need for standardized data and resource allocation policies.

This study displays several strengths. Firstly, the thoroughness of the search process is a notable asset. We meticulously scoured official sources to collect pertinent data regarding the impact of COVID-19 on six countries, representing almost 80% of the Latin American population. Secondly, the use of a systematic review to search for the best evidence as a source for calculating EM and YLL coupled with the supplementation of official databases represents another notable strength. These two outcomes offer a more comprehensive and conclusive understanding of mortality aspects, especially given the inherent challenges in analyzing mortality data in the context of an outbreak/pandemic. Finally, the cost analysis addresses a crucial aspect of the disease burden, particularly in low-middle-income countries.

This study has certain limitations. First, a limitation arises from the lack of information and heterogeneity of data recording in the countries. Nevertheless, the research team used different methods to homogenize this information. However, it is important to note that, despite these adjustments, direct comparisons between countries should be interpreted cautiously. We prioritized publicly available national datasets whenever possible. However, for certain variables, national-level data were incomplete or unavailable at the time of analysis. In these cases, we supplemented the data with published studies, selecting those with robust methodologies and representative patient populations when possible. While we acknowledge the limitations of single-center studies, they provided the best available estimates in the absence of comprehensive national statistics, allowing us to generate informed approximations where official data were lacking.

Second, in the absence of Colombian-specific data when writing, this study initially utilized Chilean data to estimate that 18% of COVID-19 hospitalized patients in Colombia required mechanical ventilation, approximating 18,000 patients. Recent analysis, informed by a Cali-based study reporting that 69% of ICU patients needed invasive ventilation [64], suggests that of an estimated 102,000 hospitalized patients by December 2020, approximately 25,500 required ICU care, with 17,850 (70% of ICU cases) needing mechanical ventilation. These figures align closely with our initial estimate, reinforcing its plausibility despite early data limitations [65].

Third, we assessed the disease burden during the dominance of Alpha and Beta variants in a predominantly unvaccinated population without addressing the spread of variants with varying degrees of severity within the region. On the other hand, the cost estimation is based on an approximation based on WHO clinical guidelines adapted by local experts, which may have the limitation of not accurately representing the reality of the countries. In addition, the cost information was extracted from different sources, which may bias the comparison between countries. However, this methodology is commonly used because of the lack of a health system resource use registry and cost-of-illness studies in the region. This shows the need for more studies examining the impact of COVID-19 on the use of healthcare resources and related costs in the adult population. Finally, YLL (and related measures) must be used carefully regarding COVID-19, a disease that primarily affects the elderly. More extensive longevity in a population results in a higher YLL, as observed in Cuba. This effect persists even with age-adjusted YLL. For illustration, consider an extreme case where half of the people over 70 years of age die from a disease. In a population with no individuals over 70 years of age, the age-adjusted YLL was 0. In contrast, the YLL was positive in the other, with a significantly older population. This effect cannot be fully mitigated with age-adjusted YLL. Nevertheless, the figures presented represent a helpful starting point. Additionally, efforts to obtain timely data from national authorities, such as the Colombian Ministry of Health, resulted in delayed access to a database that provided no further information beyond existing sources

Important policy recommendations from this study are improving data standardization and increasing investment in healthcare infrastructure, including ICU availability and overcoming medical staff wage disparities.

## 5. Conclusions

The health and economic burden of COVID-19 varied significantly across the six Latin American countries during the pre-vaccination period. This study highlights several key areas that require attention in the Latin American region. First, there is a need to standardize and improve the definitions used in health information systems. The lack of uniformity in definitions can lead to inconsistencies in data interpretation, making cross-country comparisons difficult and hindering evidence-based decision making. Second, standardizing data collection and management practices is essential. The heterogeneity in methodologies across countries and institutions poses significant challenges for regional data integration and analysis. Establishing standardized data collection and management protocols would improve comparability, facilitate collaboration, and strengthen public health responses across the region.

## Figures and Tables

**Table 1:** COVID-19 cases according to severity and incidence rates per 100,000 persons by country, sex, and age *.

Indicators	Argentina	Brazil	Chile	Colombia	Mexico	Peru
COVID-19 All cases, N (%)	3,711,250 (100)	11,956,157 (100)	728,812 (100)	3,240,355 (100)	2,363,214 (100)	1,867,034 (100)
Mild *n* (%)	3,544,077 (95)	10,459,717 (88)	675,039 * (93)	3,133,423 * (97)	1,898,410 (80)	1,768,939 (94)
Moderate and Severe *n* (%)	133,339 (4)	836,398 (7)	35,236 * (5)	69,900 * (2)	427,694 (18)	86,796 (5)
Critical *n* (%)	33,834 (1)	478,171 (4)	18,537 (2)	33,503 * (1)	37,110 (2)	11,299 (1)
Missing data	0	181,871 (1)	0	0	0	0
Incidence (both sex)	11,490	7507	4865	8684	2700	8225
18–49 years	12,362	9635	5122	8712	2486	7719
50–64 years	11,648	3559	4969	8680	3248	9789
>=65 years	7886	2968	3724	8527	3039	8696
Incidence Female	11,210	7154	4767	8852	2597	7821
18–49 years	12,520	9483	5102	9029	2466	7471
50–64 years	11,318	3220	4802	8814	3044	9154
>=65 years	6815	2518	3564	7985	2589	7754
Incidence Male	11,594	7874	4965	8507	2811	8647
18–49 years	12,035	9779	5143	8391	2506	7974
50–64 years	11,849	3933	4973	8534	3478	10,445
>=65 years	9092	3569	3922	9204	3575	9781

* From February 2020 until 1 February 2021 for Chile (12 months), 1 June 2021 for Argentina, Brazil, Colombia, Mexico, and Peru (16 months). Note: Incidence rate Missing data: Argentina (0.8–1.7%) Brazil (0.01–0.1%), Chile, Colombia, Mexico and Peru (0%). Severity based on the definition of disease severity by WHO on the Living Guidance for clinical management of COVID-19 (WHO). Missing data: Brazil (1.5%), Argentina, Chile, Colombia, Mexico and Peru (0%). * Values estimated from published studies. (Power BI Report) [40,41].

**Table 2:** COVID-19 hospitalization admission by country per sex and age, critical care admission, and hospitalized cases with mechanical ventilation *.

Indicators	Argentina	Brazil	Chile **	Colombia **	Mexico	Peru
COVID-19 All cases, N	3,711,250	11,956,157	728,812	3,240,355	2,363,214	1,867,034
Hospital admission (%)	4.5	11	7.4	3.2	19.7	5.3
Hospitalization rate (per 100,000 cases)	3562	7362	9380	469	18,096	5230
Hospitalizations both sex n (%)	167,173 (100)	1,314,569 (100)	53,773 (100)	103,402 (100)	464,804 (100)	98,095 (100)
18–49 years	50,899 (30)	388,030 (20)	11,509 (21)	29,262 (28)	127,446 (28)	36,081 (37)
50–64 years	43,678 (26)	414,813 (31)	22,426 (42)	27,768 (27)	163,738 (35)	28,063 (28)
>=65 years	72,596 (44)	511,726 (39)	19,838 (37)	46,372 (45)	173,620 (37)	33,951 (35)
Hospitalizations Female	73,366 (44)	582,555 (44.3)	28,520 (53)	44,375 (43)	187,952 (40)	44,168 (45)
18–49 years	23,766 (32)	156,044 (27)	6695 (23)	13,616 (30)	49,432 (26)	20,801 (47)
50–64 years	17,108 (23)	179,109 (31)	12,307 (43)	10,536 (24)	65,800 (35)	9988 (23)
>=65 years	32,492 (44)	247,402 (42)	9518 (34)	20,223 (46)	72,720 (39)	13,379 (30)
Hospitalizations Male	90,999 (54)	731,910 (54.4)	25,253 (47)	59,024 (57)	276,852 (60)	53,677 (55)
18–49 years	26,590 (29)	231,955 (32)	4814 (19)	15,851 (27)	78,014 (28)	15,117 (28)
50–64 years	26,214 (29)	235,675 (32)	10,120 (40)	17,514 (29)	97,938 (35)	18,018 (34)
>=65 years	38,195 (42)	264,280 (36)	10,319 (41)	25,659 (44)	100,900 (37)	20,542 (38)
Critical care admission both sex n (%)	33,834 (20)	478,171 (36)	18,537 (34)	33,503 (32)	37,113 (8)	11,299 (11)
Hospitalized cases with mechanical ventilation both sex n (%)	19,387(12)	268,411 (20)	8,386 (16)	18,174 (18)	62,640 (14)	8,576 (9)

Elaborated from Ministry of Health (MOH) databases. Notes: Missing: Argentina (0.9–2.8%), Brazil (0.01%), Peru (0.1–0.3%), Chile, Colombia and Mexico (0%). * From February 2020 until 1 February 2021 for Chile (12 months), until 1 June 2021 for Argentina, Brazil, Colombia, Mexico and Peru (16 months). ** Values estimated from published studies [28].

**Table 3:** COVID-19 death counts and percentages by country, sex and age *.

Indicators n (%)	Argentina	Brazil	Chile	Colombia	Mexico	Peru
Death both sex	92,434 (100)	521,577 (100)	18,480 (100)	89,137 (100)	237,947 (100)	184,969 (100)
18–49 years	6,816 (7)	88,035 (16.9)	1,041 (5.6)	8,928 (10)	37,678 (16)	23,947 (13)
50–64 years	19,257 (21)	140,505 (26.9)	5679 (30.7)	22,104 (25)	82,553 (35)	54,055 (29)
>=65 years	66,361 (72)	293,037 (56.2)	11,760 (63.6)	58,105 (65)	117,716 (49)	106,967 (58)
Death Female	37,714 (41)	228,360 (43.8)	6,542 (35.4)	33,978 (38)	89,116 (37)	66,805 (36)
18–49 years	2,531 (7)	35,620 (15.6)	262 (4)	2,912 (9)	12,074 (14)	7,729 (12)
50–64 years	6,507 (17)	58,070 (25.4)	2,682 (40.9)	7,834 (23)	30,249 (34)	18,360 (27)
>=65 years	28,676 (76)	134,670 (58.9)	3,598 (54.9)	23,232 (68)	46,793 (52)	40,716 (61)
Death Male	52,728 (57)	293,159 (50)	11,938 (64.6)	55,159 (62)	148,831 (63)	118,164 (64)
18–49 years	4,201 (8)	52,402 (18)	477(4)	6,016 (11)	25,604 (17)	16,218 (14)
50–64 years	12,604 (24)	82,419 (28)	4,895 (41)	14,270 (26)	52,304 (35)	35,695 (30)
>=65 years	35,923 (68)	158,338 (54)	6,566 (55)	34,873 (63)	70,923 (48)	66,251 (56)
Mortality rate per 100,000	276.1	327.5	123.4	238.8	271.8	814.9
Case fatality rate	2.5	4.4	2.5	2.8	10.1	9.9

Notes: Missing: Argentina (0.9–2.7%), Brazil (both sex: 0.01–0.02%), Chile, Colombia, Mexico and Peru (0%). * from February 2020 until 1 February 2021 for Chile (12 months), until 1 June 2021 for Argentina, Brazil, Colombia, Mexico and Peru (16 months)). Elaborated from Ministry of Health (MOH) databases.

**Table 4:** Years of life lost (YLLs) and disability-adjusted life years (DALYs) per 100,000 persons in six countries *.

Country	YLLs	YLDs	DALYs	DALYs/100,000
Argentina (min-max)	510,222 -	9235 (3418–69,900)	519,457 (513,640–580,122)	1680.6 (1661–1876)
Brazil (min-max)	3,312,346 -	59,953 (22,192–453,791)	3,372,299 (3,334,538–3,766,137)	2209 (2184–2467)
Chile (min-max)	241,089 -	4363 (1615–33,029)	245,452 (242,704–274,118)	1697.3 (1678–1895)
Colombia (min-max)	885,793 -	16,033 (5934–121,353)	901,826 (891,727–1,007,146)	2532.7 (2504–2828)
Mexico (min-max)	2,097,504 -	37,761 -	2,135,265 -	2549.5 -
Peru (min-max)	744,331 -	13,472 (4987–101,973)	757,803 (749,318–846,304)	3510.7 (3471–3920)

Notes: YLL data were retrieved from the studies shown in Table S2. All Years Lived with Disability (YLD) estimates were derived using the ratio (YLL/YLD) calculated in the study conducted by Escudero-Salinas and multiplied by the YLL of each country [29]. The Min-Max values for estimating Disability-Adjusted Life Years (DALYs) were determined by utilizing the range of values reported in a systematic review [30]; the YLDs range was calculated from the DALYs range and YLL. * Period under pre-vaccination analysis. Elaborated from Ministry of Health (MOH) databases.

**Table 5:** Cost per case of COVID-19 in USD *.

	Argentina	Brazil	Chile	Colombia	Mexico	Peru
Mild	USD 68.9 (100.0%)	USD 26.6 (100.0%)	USD 56.3 (100.0%)	USD 28.9 (100.0%)	USD 44.2 (100.0%)	USD 27.6 (100.0%)
Consultations	USD 20.0 (29.0%)	USD 7.2 (27.1%)	USD 46.5 (82.5%)	USD 13.9 (48.2%)	USD 20.5 (46.5%)	USD 13.2 (47.7%)
Diagnostic and laboratory tests	USD 45.5 (66.0%)	USD 17.6 (66.1%)	USD 9.6 (17.0%)	USD 13.1 (45.3%)	USD 21.9 (49.6%)	USD 14.1 (50.9%)
Hospitalizations	USD 0.0 (0.0%)	USD 0.0 (0.0%)	USD 0.0 (0.0%)	USD 0.0 (0.0%)	USD 0.0 (0.0%)	USD 0.0 (0.0%)
Drugs	USD 3.4 (5.0%)	USD 1.8 (6.8%)	USD 0.3 (0.6%)	USD 1.9 (6.5%)	USD 1.8 (4.0%)	USD 0.4 (1.4%)
Moderate and severe	USD 2510.0 (100.0%)	USD 1059.4 (100.0%)	USD 2971.3 (100.0%)	USD 1721.8 (100.0%)	USD 1936.6 (100.0%)	USD 1357.7 (100.0%)
Consultations	USD 9.1 (0.4%)	USD 2.8 (0.3%)	USD 24.3 (0.8%)	USD 12.2 (0.7%)	USD 12.2 (0.6%)	USD 7.8 (0.6%)
Diagnostic and laboratory tests	USD 242.3 (9.7%)	USD 77.0 (7.3%)	USD 279.4 (9.4%)	USD 448.3 (26.0%)	USD 311.0 (16.1%)	USD 199.8 (14.7%)
Hospitalizations	USD 2253.0 (89.8%)	USD 976.6 (92.2%)	USD 2667.1 (89.8%)	USD 1258.2 (73.1%)	USD 1610.0 (83.1%)	USD 1149.5 (84.7%)
Drugs	USD 5.7 (0.2%)	USD 3.0 (0.3%)	USD 0.5 (0.0%)	USD 3.1 (0.2%)	USD 3.5 (0.2%)	USD 0.6 (0.0%)
Critical	USD 23,384.2 (100.0%)	USD 19,391.5 (100.0%)	USD 19,839.8 (100.0%)	USD 7147.2 (100.0%)	USD 30,040.9 (100.0%)	USD 8053.8 (100.0%)
Consultations	USD 9.2 (0.0%)	USD 2.8 (0.0%)	USD 25.2 (0.1%)	USD 12.7 (0.2%)	USD 12.6 (0.0%)	USD 8.1 (0.1%)
Diagnostic and laboratory tests	USD 1207.7 (5.2%)	USD 351.4 (1.8%)	USD 1654.2 (8.3%)	USD 2280.8 (31.9%)	USD 1606.5 (5.3%)	USD 1032.1 (12.8%)
Hospitalizations	USD 8611.8 (36.8%)	USD 5217.6 (26.9%)	USD 9211.8 (46.4%)	USD 3806.7 (53.3%)	USD 5936.1 (19.8%)	USD 4911.5 (61.0%)
Drugs	USD 13,555.5 (58.0%)	USD 13,819.7 (71.3%)	USD 8948.7 (45.1%)	USD 1047.1 (14.7%)	USD 22,485.8 (74.9%)	USD 2102.1 (26.1%)

Source: own elaboration. * Cost reported for the year 2023.

**Table 6:** Total cost of COVID-19 by severity in USD *.

	Argentina	Brazil	Chile	Colombia	Mexico	Peru
Mild COVID cases	USD 244.2 (17.8%)	USD 278.5 (2.7%)	USD 38.0 (7.4%)	USD 90.6 (20.1%)	USD 83.8 (4.1%)	USD 48.9 (19.0%)
Moderate and severe COVID cases	USD 334.7 (24.4%)	USD 886.1 (8.5%)	USD 104.7 (20.5%)	USD 120.4 (26.7%)	USD 828.3 (40.9%)	USD 117.8 (45.7%)
Critical COVID cases	USD 791.2 (57.7%)	USD 9272.4 (88.8%)	USD 367.8 (72.0%)	USD 239.5 (53.2%)	USD 1114.8 (55.0%)	USD 91.0 (35.3%)
Total cost for all COVID cases	USD 1370.1 (100.0%)	USD 10,437.1 (100.0%)	USD 510.5 (100.0%)	USD 450.4 (100.0%)	USD 2026.9 (100.0%)	USD 257.7 (100.0%)
% of health expenditure	2.2%	5.3%	1.7%	1.5%	2.3%	1.7%

Source: own elaboration. * Cost reported for the year 2023.

## Data Availability

All data involved in this study are included in the main manuscript and its Supplementary Information Files.

## Supplementary Materials

The following supporting information can be downloaded at: https://www.mdpi.com/article/10.3390/ijerph22050669/s1, Table S1. COVID-19 data source, Table S2. General characteristics of the included studies, Table S3: characteristics of studies reporting premature mortality, Table S4. Premature mortality, YLL rates and age-adjusted YLL rates from COVID-19 for adults (>20 years) in Argentina, Table S5. Premature mortality, YLL rates and age-adjusted YLL rates from COVID-19 for adults (>20 years) in Brazil, Table S6. Premature mortality, YLL rates and age-adjusted YLL rates from COVID-19 for adults (>20 years) in Chile, Table S7. Premature mortality, YLL rates and age-adjusted YLL rates from COVID-19 for adults (>20 years) in Colombia, Table S8. Premature mortality, YLL rates and age-adjusted YLL rates from COVID-19 for adults (>20 years) in México, Table S9. Premature mortality, YLL rates and age-adjusted YLL rates from COVID-19 for adults (>20 years) in Perú, Table S10. characteristics of studies reporting overall excess deaths in six LATAM countries, Table S11. Excess deaths in six LATAM countries by sex, Table S12. Length of stay in days, Table S13. Use of resources by severity level, Table S14. Unit costs of each resource used according to the severity of COVID-19, Table S15. Sources searched for country-specific epidemiological data, Table S16. Pneumonia and Influenza average death counts, by country and age, Table S17. Costs per hospitalized case of pneumococcal and influenza in USD 2023, Table S18. Population data from 2020 by country, sex and age, Table S19A. COVID-19 cases under critical care counts and percentage by country, age, and sex, Table S19B. COVID-19 Hospitalized patients who required mechanical ventilation, counts, and percentage by country, sex, and age, Table S20. Comorbidities in COVID-19 cases by country, Table S21. COVID-19 Death counts and percentages by country, sex and age, Table S22. COVID-19 Case Fatality rate as percentage of deaths by country, sex and age, Figure S1. Flowchart for confirmed adult COVID-19 patients in Argentina, Figure S2. Flowchart for confirmed adult COVID-19 patients in Brazil, Figure S3. Flowchart for confirmed adult COVID-19 patients in Chile, Figure S4. Flowchart for confirmed adult COVID-19 patients in Colombia, Figure S5. Flowchart for confirmed adult COVID-19 patients in Mexico, Figure S6. Flowchart for confirmed adult COVID-19 patients in Perú, Figure S7. Flow chart of included studies, Figure S8. Monthly COVID-19 case fatality rate. References [18,31,32,33,34,35,42,43,44,45,46,47,48,49,59,61,62,66,67,68,69,70,71,72,73,74,75,76,77,78,79,80,81,82,83,84,85,86,87,88] are cited in the supplementary materials.
