# Supplementary material for: COVID-19 Disease and Economic Burden to Healthcare Systems in Adults in Six Latin American Countries Before Nationwide Vaccination Program: Ministry of Health Database Assessment and Literature Review
Source: Int J Environ Res Public Health. 2025 Apr 24;22(5):669. doi: 10.3390/ijerph22050669 (PMC12111093; doi:10.3390/ijerph22050669)
Supplement: Supplementary file 1 [file ijerph-22-00669-s001.zip › ijerph-3424252-supplementary.pdf]

Supplementary Material

1. COVID-19 Data and Flowcharts in Latin America **3**
Table S1. COVID-19 data source 3
Figure S1. Flowchart for confirmed adult COVID-19 patients in Argentina 4
Figure S2. Flowchart for confirmed adult COVID-19 patients in Brazil 4
Figure S3. Flowchart for confirmed adult COVID-19 patients in Chile 5
Figure S4. Flowchart for confirmed adult COVID-19 patients in Colombia 5
Figure S5. Flowchart for confirmed adult COVID-19 patients in Mexico 6
Figure S6. Flowchart for confirmed adult COVID-19 patients in Perú 6
2. Literature search strategy **7**
Inclusion and exclusion criteria 7
Design of studies included 7
Risk of bias 7
Figure S7. Flow chart of included studies. 16
Table S2. General characteristics of the included studies 16
3. Years of life lost estimation. **17**
Table S3: characteristics of studies reporting premature mortality 19
Table S4. Premature mortality, YLL rates and age-adjusted YLL rates from COVID-19 for adults (>20 years) in Argentina 20
Table S5. Premature mortality, YLL rates and age-adjusted YLL rates from COVID-19 for adults (>20 years) in Brazil 20
Table S6. Premature mortality, YLL rates and age-adjusted YLL rates from COVID-19 for adults (>20 years) in Chile .21
Table S7. Premature mortality, YLL rates and age-adjusted YLL rates from COVID-19 for adults (>20 years) in Colombia 22
Table S8. Premature mortality, YLL rates and age-adjusted YLL rates from COVID-19 for adults (>20 years) in México 22
Table S9. Premature mortality, YLL rates and age-adjusted YLL rates from COVID-19 for adults (>20 years) in Perú...23
4. Excess mortality estimation **24**
Table S10. characteristics of studies reporting overall excess deaths in six LATAM countries. 24
Table S11. Excess deaths in six LATAM countries by sex. 25
5. Length of stay in hospitalization **26**
Table S12. Length of stay in days 27
Table S13. Use of resources by severity level 27
6. Unit resource costs by severity of COVID-19 .... **28**
Table S14. Unit costs of each resource used according to the severity of COVID-19 29
7. Other vaccine-preventable diseases **30**
Table S15. Sources searched for country-specific epidemiological data. 30
Table S16. Pneumonia and Influenza average death counts, by country and age. 31
8. Search strategy for information on costs of immune preventable diseases **32**
Pubmed 32
Pneumococcal 32
Influenza 32
Lilacs 33
Table S17. Costs per hospitalized case of pneumococcal and influenza in USD 2023 33
9. Population data by country **34**
Table S18. Population data from 2020 by country, sex and age 34
Table S19A. COVID-19 cases under critical care counts and percentage by country, age, and sex. 34
Table S19B. COVID-19 Hospitalized patients who required mechanical ventilation, counts, and percentage by country, sex, and age. 35
Table S20. Comorbidities in COVID-19 cases by country. 35
Table S21. COVID-19 Death counts and percentages by country, sex and age. 36

Table S22. COVID-19 Case Fatality rate as percentage of deaths by country, sex and age. 37
Figure S8. Monthly COVID-19 case fatality rate. 37
References 38

1. COVID-19 Data and Flowcharts in Latin America

Table S1. COVID-19 data source

Country	Data source	Link
Argentina	MOH; DEIS	https://www.argentina.gob.ar/salud/deis; http://www.datos.salud.gob.ar/
Brazil	MOH	https://opendatasus.saude.gov.br/dataset
Chile	MOH; DEIS	https://www.minsal.cl/; https://deis.minsal.cl/
Colombia	MOH; DANE	https://www.datos.gov.co/; https://www.dane.gov.co/
México	RENAPO, INEGI	https://www.gob.mx/segob/renapo; https://www.inegi.org.mx/
Perú	MOH; INEI	https://www.minsa.gob.pe/defunciones/; https://www.gob.pe/inei/

MOH: Ministry of Health; DEIS: Directorate of Health Statistics and Information; DANE: National Administrative Department of Statistics; RENAPO: General Directorate of the National Registry of Population and Identity; INEGI: National Institute of Statistic and Geography; INEI: National Institute of Statistics and Informatics.

Notes: For data regarding hospital admissions, deaths, the severity of cases, and the total incidence of COVID-19 we explored ministerial reports (hyperlinks provided in the aforementioned table). The dataset was refined through temporal and age-based filtering, with missing values meticulously recorded in the corresponding table footnotes.

Figure S1. Flowchart for confirmed adult COVID-19 patients in Argentina

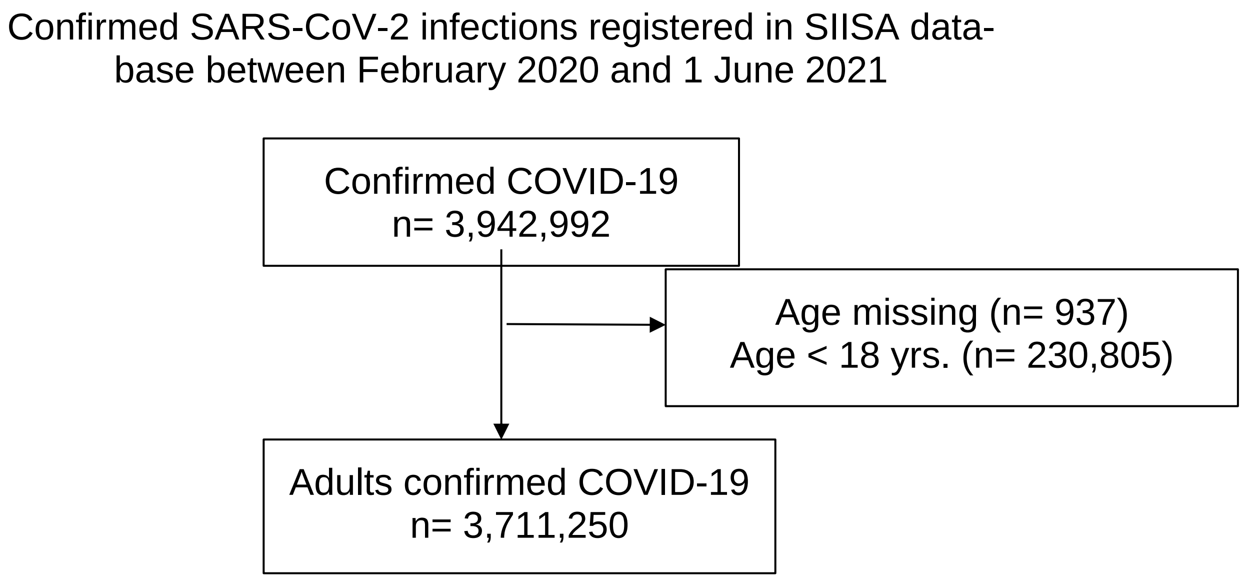

SIISA: Sistema Integrado de Información Sanitaria Argentina.

Figure S2. Flowchart for confirmed adult COVID-19 patients in Brazil

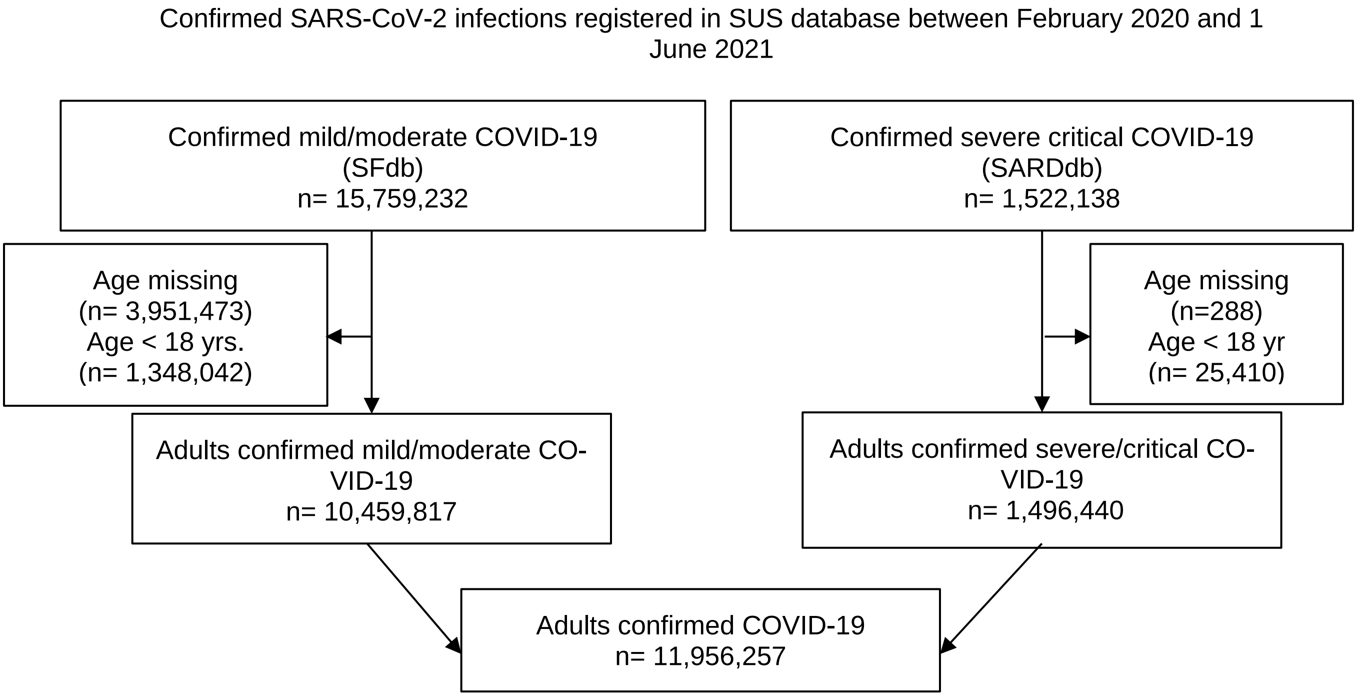

SUS: Sistema Único de Salud; SFbd: Syndromic flu database; SARDbd: Severe acute respiratory disease database.

Figure S3. Flowchart for confirmed adult COVID-19 patients in Chile

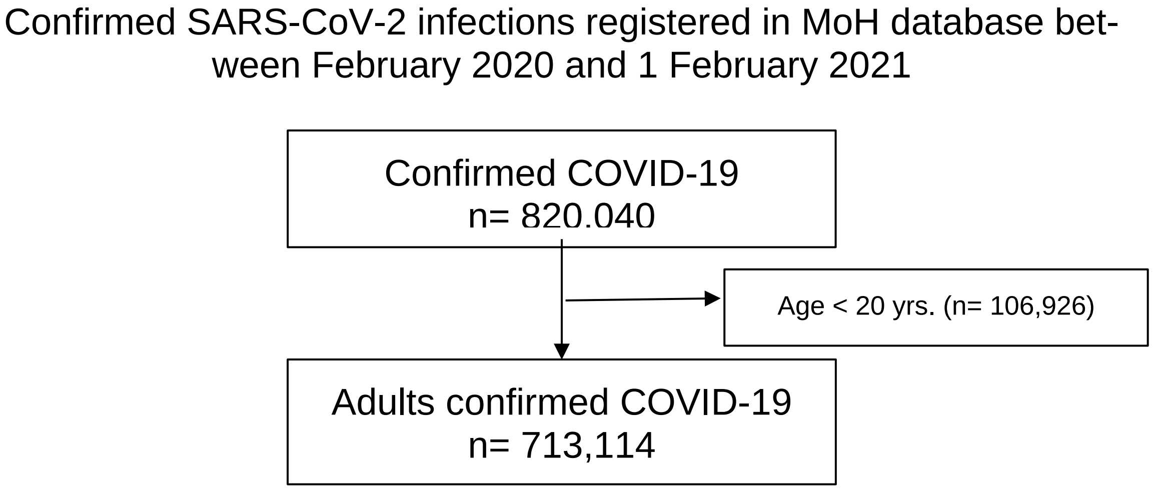

MOH: ministry of health

Figure S4. Flowchart for confirmed adult COVID-19 patients in Colombia

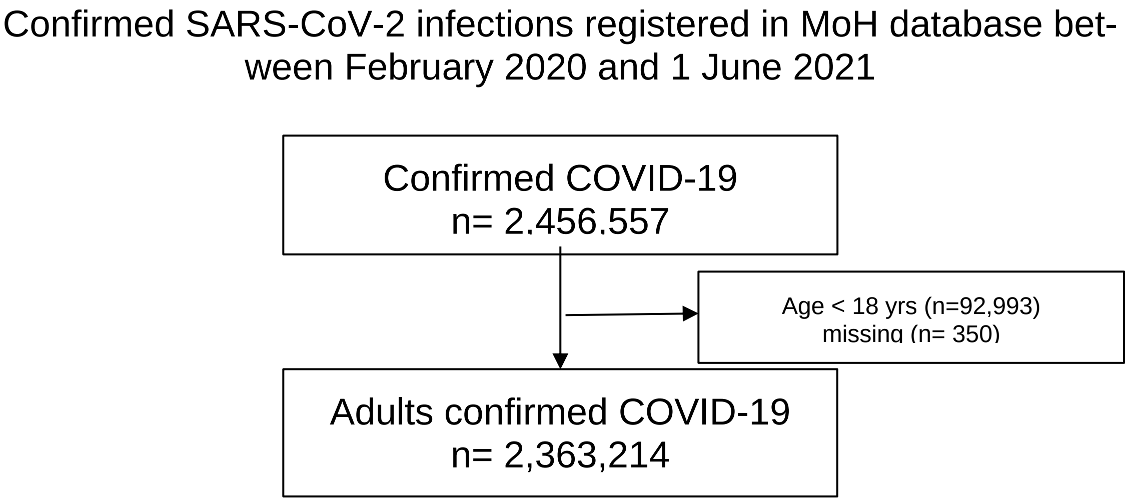

MOH: ministry of health

Figure S5. Flowchart for confirmed adult COVID-19 patients in Mexico

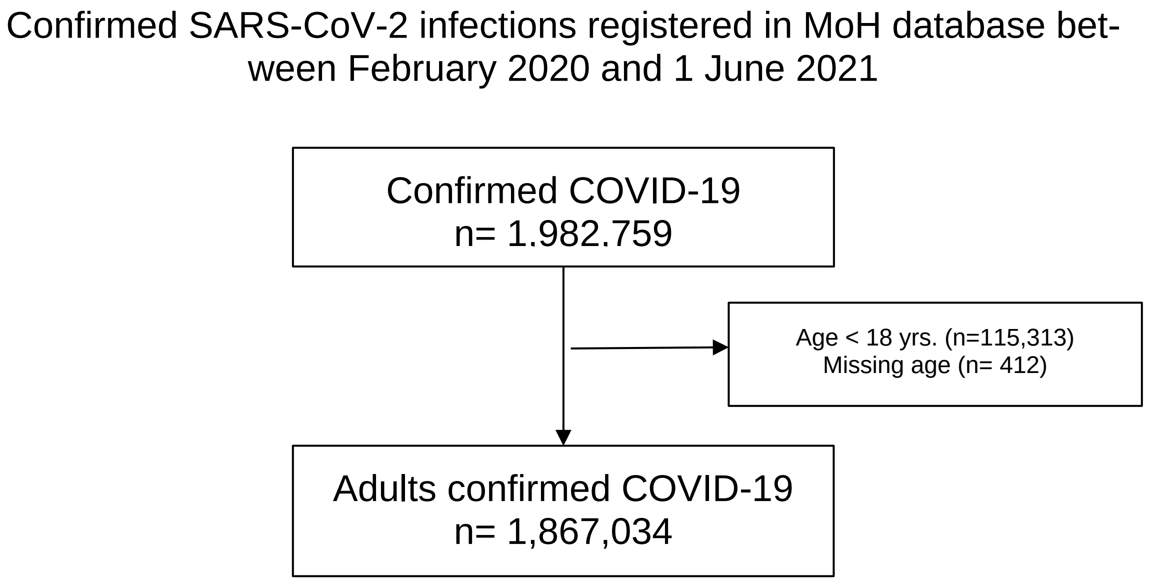

MOH: ministry of health

Figure S6. Flowchart for confirmed adult COVID-19 patients in Perú

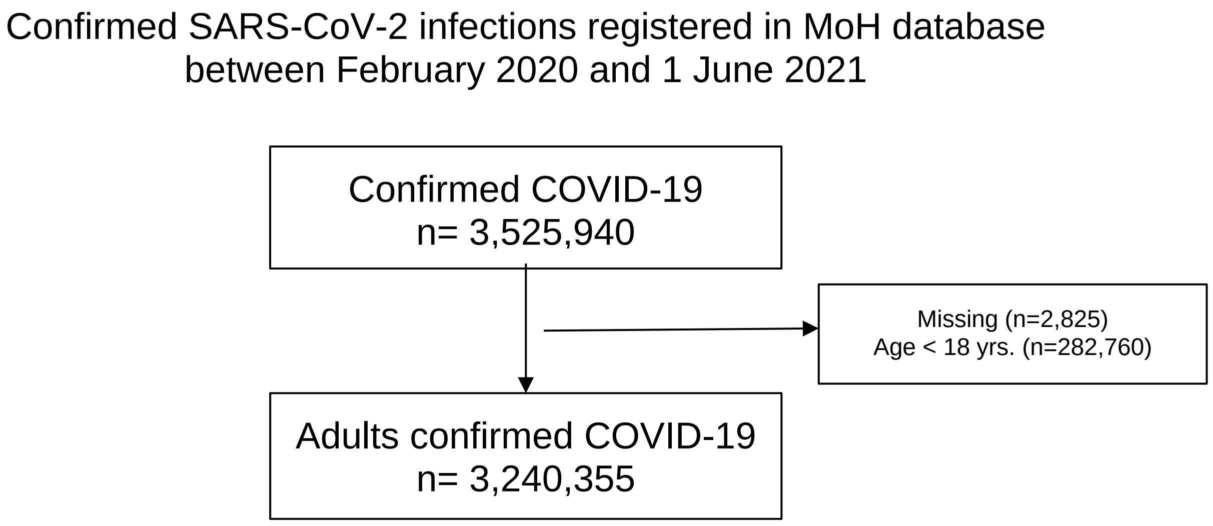

MOH: Ministry of Health

2. Literature search strategy

Inclusion and exclusion criteria

This analysis only included studies that provided data from the adult general population (≥18 years) diagnosed with COVID-19 from Argentina, Brazil, Chile, Colombia, Mexico, and Peru in the pre-vaccination period. We searched data and stratified the population by sex (female-male) and age (18 to 49 years old, 50 to 64 years old, and ≥ 65 years old). Also, we searched data on individuals with certain comorbidities (hypertension, diabetes, obesity, chronic kidney disease, asthma, chronic obstructive pulmonary disease, cardiovascular disease, and other forms of immunosuppression). Regarding types of studies, we included surveillance studies, prevalence cohorts, ministerial reports, WHO reports, budget impact analysis, and economic or direct cost evaluations with a microeconomic approach. Randomized clinical trial studies were excluded from this study. We also excluded reports from Chile after February 1st, 2021, and reports from Argentina, Brazil, Colombia, México, and Perú after June 1st, 2021, to comply with the established pre-vaccination period.

Design of studies included

We included studies of any epidemiological or economic design (whose full text was in Spanish, English, or Portuguese). Epidemiological relevant surveillance reports for the chosen calendar period were also assessed. We also included observational studies such as cohorts, case-control, case series, economic evaluations, and cost or budget impact studies. Study selection was carried out using COVIDENCE, a web-based platform designed for the systematic review process. The authors of the articles were contacted when it was necessary to obtain missing or supplementary information. Previously piloted in five studies, a predesigned general data extraction form was used. Disagreements were resolved by consensus of the entire team.

Risk of bias

The risk of bias in observational studies was assessed using a checklist developed by the US National Heart, Lung, and Blood Institute, which classifies studies as high risk of bias (Poor), uncertain (Fair), and low risk of bias (Good). For the assessment of cohort studies and cross-sectional studies, the tool comprises 14 items, while nine items apply to case series studies. The assessment was carried out independently by peer reviewers from the research team. Discrepancies were resolved by consensus of the entire team.

Several potential limitations of the literature review include incomplete data in published studies and MOH reports, heterogeneity and quality of available data, and publication bias due to the inclusion of only one geographical region in the review.

PubMed (up to 28/12/2022)

Search	Query	Results
#42	#21 AND #26 AND #40 AND #41	1,794
#41	(Argentina[Mesh] OR Argentin*[tiab] OR Argentina[pl] OR Argentin*[ad] OR Brazil[Mesh] OR Brazil*[tiab] OR Brazil[pl] OR Brazil[ad] OR Brasil*[ad] OR Colombia[Mesh] OR Colombia*[tiab] OR Colombia[pl] OR Colombia*[ad] OR Chile[Mesh] OR Chile*[tiab] OR Chile[pl] OR Chile*[ad] OR Peru[Mesh] OR Peru*[tiab] OR Peru[pl] OR Peru*[ad] OR Mexico[Mesh] OR Mexic*[tiab] OR Mexico[pl] OR Mexic*[ad])	1,143,096
#40	#27 OR #28 OR #29 OR #30 OR #31 OR #32 OR #33 OR #34 OR #35 OR #36 OR #37 OR #38 OR #39	2,853,636
#39	Fatality Rate*[tiab]	13,555
#38	Death Rate*[tiab]	25,728
#37	Mortalit*[tiab]	951,197
#36	Mortality[Majr]	71,142
#35	Reproduction Number*[tiab]	4,217
#34	Reproductive Number*[tiab]	1,277
#33	Basic Reproducti*[tiab]	3,612
#32	Person-Time Rate*[tiab]	9
#31	Attack Rate*[tiab]	5,268
#30	Incidence*[tiab]	902,355
#29	Prevalen*[tiab]	930,542
#28	Morbidit*[tiab]	463,498
#27	Morbidity[Mesh]	639,156
#26	#22 OR #23 OR #24 OR #25	8,899,229
#25	Elder*[tiab]	301,509
#24	Aged[tiab]	700,012
#23	Adult*[tiab]	1,503,628
#22	Adult[Mesh]	7,875,028
#21	#1 OR #2 OR #3 OR #4 OR #5 OR #6 OR #7 OR #8 OR #9 OR #10 OR #11 OR #12 OR #13 OR #14 OR #15 OR #16 OR #17 OR #18 OR #19 OR #20	340,036
#20	Ncov*[tiab]	3,396
#19	N-Cov*[tiab]	117
#18	CV-19*[tiab]	83
#17	CV19*[tiab]	34
#16	HCov*[tiab]	1,572
#15	Corono Virus[tiab]	2

#14	Virus Corona[tiab]	4,520
#13	Coronovir*[tiab]	80
#12	Coronavir*[tiab]	121,125
#11	(Pneumonia[tiab] AND Wuhan[tiab] AND 2019[tiab])	1,343
#10	SARSCoV2[tiab]	91,791
#9	SARS-CoV2[tiab]	4,055
#8	SARS-CoV-2[tiab]	106,031
#7	COVID19*[tiab]	270,445
#6	COVID-19[tiab]	283,395
#5	Corona Virus[tiab]	3,446
#4	COVID-19 Vaccines[Mesh]	18,102
#3	COVID-19[Mesh]	199,414
#2	SARS-CoV-2[Mesh]	144,085
#1	Coronavirus[Mesh]	157,610

EMBase (Ovid) 28/12/2022

Embase <1974 to 2022 December 27>

1 exp Coronavirinae/ 105003

2 exp Severe acute respiratory syndrome coronavirus 2/ 82609

3 exp coronavirus disease 2019/ 287563

4 exp SARS-CoV-2 vaccine/ 28493

5 (Coron* adj1 Virus).ti,ab. 4099

6 COVID-19.ti,ab. 297576

7 COVID19*.ti,ab. 5354

8 SARS-CoV-2.ti,ab. 102686

9 SARS-CoV2.ti,ab. 5366

10 SARSCoV2.ti,ab. 384

11 (Pneumonia and Wuhan and "2019").ti,ab. 1461

12 Coronavir*.ti,ab. 113956

13 Coronovir*.ti,ab. 98

14 HCov*.ti,ab. 1626

15 CV19*.ti,ab. 65

16 CV-19*.ti,ab. 214

17 N-Cov*.ti,ab. 156

18 Ncov*.ti,ab. 3221

19	or/1-18	390291
20	exp adult/	10205265
21	Adult*.ti,ab.	1974867
22	Aged.ti,ab.	940568
23	Elder*.ti,ab.	414753
24	or/20-23	11270288
25	exp morbidity/	416712
26	Morbidit*.ti,ab.	699037
27	Prevalen*.ti,ab.	1314693
28	Incidence*.ti,ab.	1295130
29	(Attack adj1 Rate*).ti,ab.	6031
30	(Person-Time adj1 Rate*).ti,ab.	15
31	(Basic adj1 Reproducti*).ti,ab.	3179
32	(Reproductive adj1 Number*).ti,ab.	1182
33	(Reproduction adj1 Number*).ti,ab.	3667
34	mortality/	863051
35	Mortalit*.ti,ab.	1381678
36	(Death adj1 Rate*).ti,ab.	32825
37	(Fatality adj1 Rate*).ti,ab.	16270
38	or/25-37	4082478
39	exp Argentina/ or Argentin*.mp.	38045
40	exp Brazil/ or Brazil*.mp. or Brasil*.mp.	218433
41	exp Colombia/ or Colombia*.mp.	32265
42	exp Chile/ or Chile*.mp.	29549
43	exp Peru/ or Peru*.mp.	26973
44	exp Mexico/ or Mexic*.mp.	101605
45	or/39-44	421583
46	19 and 24 and 38 and 45	2223

EconLIT (Ovid) 28/12/2022

Econlit <1886 to December 15, 2022>

1	(Coron* adj1 Virus).ti,ab.	38
2	COVID-19.ti,ab.	8630
3	COVID19*.ti,ab.	79
4	SARS-CoV-2.ti,ab.	178

5	SARS-CoV2.ti,ab.	6
6	SARSCoV2.ti,ab.	0
7	(Pneumonia and Wuhan and "2019").ti,ab.	1
8	Coronavir*.ti,ab.	1294
9	Coronovir*.ti,ab.	0
10	HCov*.ti,ab.	0
11	CV19*.ti,ab.	0
12	CV-19*.ti,ab.	0
13	N-Cov*.ti,ab.	3
14	Ncov*.ti,ab.	9
15	or/1-14	9135
16	Adult*.ti,ab.	11137
17	Aged.ti,ab.	5066
18	Elder*.ti,ab.	5411
19	or/16-18	19852
20	Morbidit*.ti,ab.	1093
21	Prevalen*.ti,ab.	9626
22	Incidence*.ti,ab.	9955
23	(Attack adj1 Rate*).ti,ab.	13
24	(Person-Time adj1 Rate*).ti,ab.	0
25	(Basic adj1 Reproducti*).ti,ab.	30
26	(Reproductive adj1 Number*).ti,ab.	11
27	(Reproduction adj1 Number*).ti,ab.	71
28	Mortalit*.ti,ab.	9719
29	(Death adj1 Rate*).ti,ab.	578
30	(Fatality adj1 Rate*).ti,ab.	186
31	or/20-30	29222
32	(Argentin* or Brazil* or Brasil* or Colombia* or Chile* or Peru* or Mexic*).mp.	56626
33	15 and 19 and 31 and 32	1

LILACS (BVS Eng) 28/12/2022

(MH Coronavirus OR MH SARS-CoV-2 OR MH COVID-19 OR MH COVID-19 Vaccines OR Corona OR COVID-19$ OR COVID19$ OR SARS-CoV-2$ OR SARS-CoV2$ OR SARSCoV2$ OR Coronavir$ OR Coronovir$ OR Corono OR HCov$ OR CV19$ OR CV-19$ OR N-Cov$ OR Ncov$) AND (MH Adult OR Adult$ OR Aged OR Ancian$ OR Idoso$ OR Elder$ OR Ancião$) AND (MH Morbidity OR Morbid$ OR Prevalen$ OR Incidenc$ OR ((Atack OR Ataque OR Death OR Muerte OR Morte) AND (Rate OR Tasa OR Taxa)) OR ((Reproduc$) AND (Number OR Número)) OR MH Mortality OR Mortali$) AND (MH Argentina OR Argentin$ OR MH Brazil OR Brazil$ OR Brasil$ OR MH Colombia OR Colombia$ OR MH Chile OR Chile$ OR MH Peru OR Peru$ OR MH Mexico OR Mexic$) [Words]

678

CINAHL (EBSCO) 29/12/2022

#	Query	Results
S45	S18 AND S23 AND S37 AND S44	399
S44	S38 OR S39 OR S40 OR S41 OR S42 OR S43	308,567
S43	(MH "Mexico") OR TX Mexico*	136,272
S42	(MH "Peru") OR TX Peru*	27,602
S41	(MH "Chile") OR TX Chile*	21,957
S40	(MH "Colombia") OR TX Colombia*	19,289
S39	(MH "Brazil") OR TX Brazil* OR TX Brasil*	164,351
S38	(MH "Argentina") OR TX Argentin*	19,435
S37	S24 OR S25 OR S26 OR S27 OR S28 OR S29 OR S30 OR S31 OR S32 OR S33 OR S34 OR S35 OR S36	743,786
S36	TI (Fatality N1 Rate*) OR AB (Fatality N1 Rate*)	3,062
S35	TI (Death N1 Rate*) OR AB (Death N1 Rate*)	8,002
S34	TI Mortalit* OR AB Mortalit*	240,562
S33	(MM "Mortality")	14,696
S32	TI (Reproduction N1 Number*) OR AB (Reproduction N1 Number*)	461
S31	TI (Reproductive N1 Number*) OR AB (Reproductive N1 Number*)	207
S30	TI (Basic N1 Reproducti*) OR AB (Basic N1 Reproducti*)	313
S29	TI (Person-Time N1 Rate*) OR AB (Person-Time N1 Rate*)	14
S28	TI (Attack N1 Rate*) OR AB (Attack N1 Rate*)	1,118
S27	TI Incidence* OR AB Incidence*	203,032
S26	TI Prevalen* OR AB Prevalen*	265,943
S25	TI Morbidit* OR AB Morbidit*	113,971
S24	(MH "Morbidity+")	192,285
S23	S19 OR S20 OR S21 OR S22	2,307,242
S22	TI Elder* OR AB Elder*	115,476
S21	TI Aged OR AB Aged	232,644
S20	TI Adult* OR AB Adult*	449,261
S19	(MH "Adult+")	2,047,089
S18	S1 OR S2 OR S3 OR S4 OR S5 OR S6 OR S7 OR S8 OR S9 OR S10 OR S11 OR S12 OR S13 OR S14 OR S15 OR S16 OR S17	116,167
S17	TI N-Cov* OR AB N-Cov*	6

S16	TI CV19* OR- AB CV-19*	0
S15	TI CV19* OR AB CV19*	3
S14	TI HCov* OR AB HCov*	132
S13	TI Coronovir* OR AB Coronovir*	21
S12	TI Coronavir* OR AB Coronavir*	27,792
S11	TI (Pneumonia AND Wuhan AND 2019) OR AB (Pneumonia AND Wuhan AND 2019)	272
S10	TI SARSCoV2* OR AB SARSCoV2*	21
S9	TI SARS-CoV2* OR AB SARS-CoV2*	574
S8	TI SARS-CoV-2* OR AB SARS-CoV-2*	16,510
S7	TI COVID19* OR AB COVID19*	705
S6	TI COVID-19 OR AB COVID-19	98,822
S5	TI (Coron* N1 Virus) OR AB (Coron* N1 Virus)	744
S4	(MH "COVID-19 Vaccines")	5,289
S3	(MH "COVID-19+")	38,257
S2	(MH "SARS-CoV-2")	1,215
S1	(MH "Coronavirus+")	2,991

Web of Science 29/12/2022

1,824 results from Web of Science Core Collection for:

(TS=Coronavirus OR TS=SARS-CoV-2 OR TS=COVID-19 OR TS=COVID-19 Vaccines OR TI=(Coron NEAR/1 Virus) OR AB=(Coron* NEAR/1 Virus) OR TI=COVID-19 OR AB=COVID-19 OR TI=COVID19* OR AB=COVID19* OR TI=SARS-CoV-2 OR AB=SARS-CoV-2 OR TI=SARS-CoV2 OR AB=SARS-CoV2 OR TI=SARSCoV2 OR AB=SARSCoV2 OR TI=(Pneumonia AND Wuhan AND 2019) OR AB=(Pneumonia AND Wuhan AND 2019) OR TI=Coronavir* OR AB=Coronavir* OR TI=(Coron* NEAR/1 Virus) OR AB=(Coron* NEAR/1 Virus) OR TI=Coronovir* OR AB=Coronovir* OR TI=HCov* OR AB=HCov* OR TI=CV19* OR AB=CV19* OR TI=CV-19* OR AB=CV-19* OR TI=N-Cov* OR AB=N-Cov* OR TI=NCov OR AB=NCov) AND (TS=Adult OR TI=Adult* OR AB=Adult* OR TI=Aged OR AB=Aged OR TI=Elder* OR AB=Elder*) AND (TS=Morbidity OR TI=Morbidit* OR AB=Morbidit* OR TI=Prevalen* OR AB=Prevalen* OR TI=Incidence* OR AB=Incidence* OR TI=(Attack NEAR/1 Rate*) OR AB=(Attack NEAR/1 Rate*) OR TI=(Person-Time NEAR/1 Rate*) OR AB=(Person-Time NEAR/1 Rate*) OR TI=(Basic NEAR/1 Reproducti*) OR AB=(Basic NEAR/1 Reproducti*) OR TI=(Reproductive NEAR/1 Number*) OR AB=(Reproductive NEAR/1 Number*) OR TI=(Reproduction NEAR/1 Number*) OR TS=Mortality OR TI=Mortalit* OR AB=Mortalit* OR TI=(Death NEAR/1 Rate*) OR AB=(Death NEAR/1 Rate*) OR TI=(Fatality NEAR/1 Rate*) OR AB=(Fatality NEAR/1 Rate*)) AND (TS=Argentina OR TI=Argentin* OR AB=Argentin* OR CU=Argentina OR OG=Argentin* OR TS=Brazil OR TI=Brazil* OR AB=Brazil* OR CU=Brazil OR OG=Brazil OR OG=Brasil* OR TS=Colombia OR TI=Colombia* OR AB=Colombia* OR CU=Colombia OR OG=Colombia* OR TS=Chile OR TI=Chile* OR AB=Chile* OR CU=Chile OR OG=Chile* OR TS=Peru OR TI=Peru* OR AB=Peru* OR CU=Peru OR OG=Peru* OR TS=Mexico OR TI=Mexic* OR AB=Mexic* OR CU=Mexico OR OG=Mexic*

Figure S7. Flow chart of included studies.

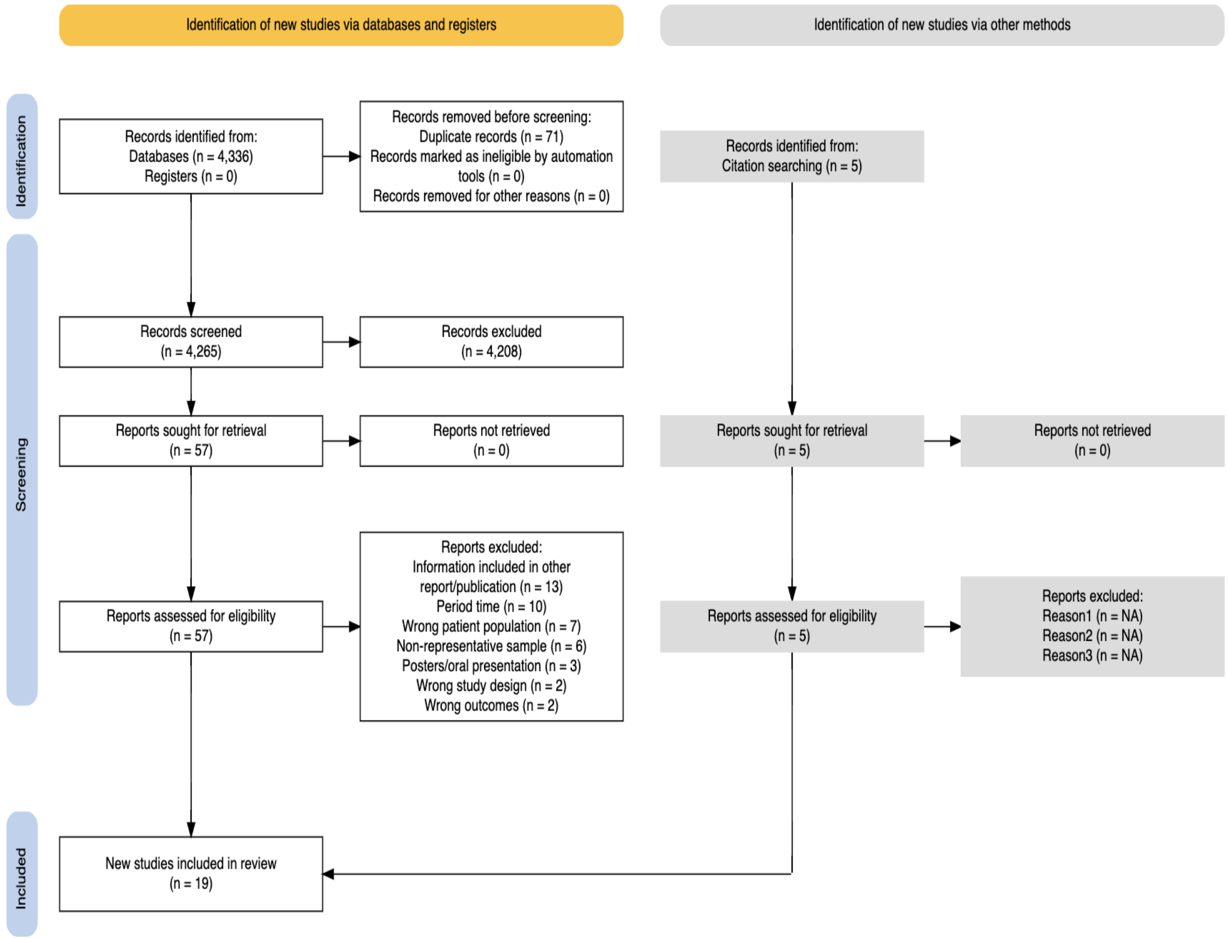

Table S2. General characteristics of the included studies

Included studies	Country	Period of analysis	Data Source	Study design	Impact metrics	Risk of bias
Pifarré I Arolas H et al. (2021)[1]	Multinational	9 months	COVerAGE-DB	Observational	YLLs	Fair
Castro APB et al. (2021)[2]	Brazil	12 months	Ministério da Saúde	Observational	PYLLs	Good
Islam N et al. (2021)[3]	Chile	12 months	Human Mortality Database	Time series analysis	YLLs	Fair
Ugarte MP et al. (2022)[4]	Multinational	8 months	Instituto Nacional de Salud	Observational	PYLLs	Fair
Salinas-Escudero G et al. (2021)[5]	Mexico	10 months	Ministerio de Salud de México	Observational	YLLs	Fair
Rearte A et al. (2021)[6]	Argentina	12 months	DEIS y Registro Civil	Observational	Excess mortality and *P*-score	Fair
dos Santos AM et al. (2021)[6,7]	Brazil	12 months	Ministério da Saúde	Observational	Excess mortality and *P*-score	Fair
Quapper DD et al. (2021)[8]	Chile	5 months	DEIS	Observational	Excess mortality and *P*-score	Fair
Benavides GF et al (2022)[9]	Multinational	11 months	DANE	Observational	Excess mortality and *P*-score	Fair
Palacio-Mejía LS et al. (2021)[10]	Mexico	12 months	INEGI, RENAPO	Observational	Excess mortality and *P*-score	Good
Huarcaya WV et al (2022)[11]	Perú	18 months	SINADEF-INEI	Observational	Excess mortality and *P*-score	Fair
Araujo M et al. (2020)[12]	Chile	4 months	Hospital records	Observational	Hospital mortality	Good
Lima EEC et al. (2021)[13]	Multinational	12 months	Ministry of Health	Observational	Excess mortality and *P*-score	Fair
Macchia A et al. (2021)[14]	Argentina	6 months	Integrated Health Information System (SIISA)	Observational	Incidence rate	Fair
Machado-Alba JE et al. (2021)[15]	Colombia	5 months	Hospital records	Observational	Hospital mortality	Fair
Ramos, D. A et al. (2022)[16]	Colombia	8 months	Hospital records	Observational	Hospital mortality	Fair
Schonfeld D et al. (2021)[17]	Argentina	7 months	Integrated Health Information System (SIISA)	Observational	Case Fatality Rate	Good
Sorensen, RJD et al. (2022)[18]	Multinational	18 months	Ministry of Health	Observational	Excess mortality	Good
Riquelme JD et al. (2022)[19]	Chile	8 months	Hospital records	Observational	Hospital mortality	Fair

Notes: DEIS: Dirección de estadísticas e información de la Salud; DANE: Departamento administrativo nacional de estadística; INEGI: Instituto nacional de Estadística y Geografía; RENAPO: Registro nacional de población; INEI: Instituto nacional de Estadística e Informática; SINADEF: sistema informático nacional de defunciones; M: male; F: female; N/R: not reported.

3. Years of life lost estimation.

In the systematic review performed, various studies were identified, and those with the broadest analysis period (considering our study's period of interest) and reported results of total and sex-disaggregated absolute years of life lost (YLL) were prioritized for this report. We estimate the YLL rates as they account for the population size.[1] For population estimation in specific ages, we use data from the Department of Economics and Social Affairs from the United Nations.[2]

The inherent limitations of all-ages YLL rates can be overcome by utilizing the direct age-standardization method, which involves the application of a standard population.[3] Through the calculation of age-standardized YLL rates (ASYR), adjustments can be made for differences in population age distribution, thus facilitating comparisons between groups with dissimilar population age structures.[1] The ASYR can be calculated by the formula:

$$ASYR\ (c,s,t) = \sum_{a=18}^{105} YLL\ rate\ (c,s,a,t) * W\ (a)$$

where YLL rate (c, s, a, t) is the YLL rate due to cause c, in the population of sex s and age a, and period t multiplied by W(a), which is the standard WHO population weight at age a.[3] In order to calculate the ASYR, the absolute numbers of YLL for each age group are necessary. Although the total crude YLL data are reported in the literature for each country, age-disaggregated data are not available for Argentina, Brazil, Chile, Colombia, and Peru. Consequently, the proportional distribution of the absolute number of years of YLL was estimated from the study reported by Salinas-Escudero et al. in Mexico.[4] This was utilized to compute the age-standardized YLL rates (YLL/100,000) in each specific age group for countries where data on age-specific absolute YLL are not readily available. In estimating the overall ASYR, we opted to retain the age categorization described in the study by Salinas-Escudero et al.[4] for individuals aged 20 years and above, given that the relative contribution of subjects aged 18-19 years represents a mere 2%, which we deemed not significant. However, to address the data gap for Argentina, Brazil, and Peru, a sensitivity analysis was conducted using the percentage distribution of raw YLL estimated from ministerial databases.

Table S3: characteristics of studies reporting premature mortality

Study	Country	Period	Data source	LE table	Overall impact metrics	Female impact metrics	Male impact metrics
Pifarré I Arolas H et al. (2021)[1]	Argentina	9 months	COVerAGE-DB	GBD-2017	aYLL: 510,222 YLL rates: 1,172.8	aYLL: 220,298	aYLL: 220,298
Pifarré I Arolas H et al. (2021)[1]	Brazil	10 months	COVerAGE-DB	GBD-2017	aYLL: 3,312,346 YLL rates: 1,593.2	aYLL: 1,479,178	aYLL: 1,833,168
Castro APB et al. (2021)[2]	Brazil	12 months	COVerAGE-DB	Up to 70 years	YPLL: 1,280,839	YPLL: 774,766	YPLL: 506,073
Islam N et al. (2021)[3]	Chile	12 months	Human Mortality Database	GBD-2010	YLL rates: 14,500	YLL rate: 11,500	YLL rate: 17,600
Pifarré I Arolas H et al. (2021)[1]	Chile	9 months	COVerAGE-DB	GBD-2017	aYLL: 241,089	aYLL: 102,009	aYLL: 139,080

					YLL rates: 1,241.5		
Pifarré I Arolas H et al. (2021)[1]	Colombia	11 months	COVerAGE-DB	GBD-2017	aYLL: 885,793<br>YLL rates: 1,773	aYLL: 321,718	aYLL: 564,075
Ugarte MP et al. (2022)[4]	Colombia	8 months	Instituto Nacional de Salud	Up to 80 years	YPLL: 293,930	YPLL: 98,526	YPLL: 195,424
Salinas-Escudero G et al. (2021)[3,5]	México	10 months	Ministerio de Salud de México	GBD-2010	aYLL: 2,126,222<br>YLL rates: 1,663.8	aYLL: 983,957*	aYLL: 1,617,845*
Pifarré I Arolas H et al. (2021)[1]	Peru	11 months	COVerAGE-DB	GBD-2017	aYLL: 744,331<br>YLL rates: 2,319.7	aYLL: 248,297	aYLL: 496,034
Ugarte MP et al. (2022)[4]	Peru	8 months	Plataforma nacional de datos abiertos	Up to 80 years	YPLL-T: 441,157	YPLL: 122,412	YPLL: 318,745
YLL: years of life lost; PYLL: potential years of life lost; M: male; F: female; GBD: Global Burden of Diseasse; YLL/death: average number of years of life lost per death; LE: life expectancy.<br>*Data estimated from Pifarré I Arolas H et al. [5]							

Table S4. Premature mortality, YLL rates and age-adjusted YLL rates from COVID-19 for adults (>20 years) in Argentina

Age group	Population	Crude YLL weight*	Absolute YLL**	YLL rate†	Std. Popula-tion weight	Age-standardized YLL
20-29	7,012,522	0.0220	25,453.5	363	0.1615	58.6
30-39	6,509,587	0.0550	63,633.8	977.5	0.1476	144.3
40-49	5,774,178	0.1190	137,680.3	2,384.4	0.1263	301.2
50-59	4,413,450	0.2090	241,808.3	5,478.9	0.0992	543.5
60-69	3,581,985.5	0.2670	308,913.1	8,624.1	0.0668	576.1
70-79	2,336,867.5	0.2090	241,808.4	10,347.5	0.0373	386
> 80	1,279,947.5	0.1180	136,523.4	10,666.3	0.0154	164.3
Total	30,908,537.5	-	1,155,820.8	38,841.8	-	2,173.9

YLL: years of life lost; Std.: standard.
*Distribution of absolute YLL across age groups estimated from Argentinean Ministry of Health COVID-19 datasets.[14]
**We assume that the cumulative absolute YLL exhibit a distribution of 98.5% for adults over 20 years old[4].
†Expressed per 100.000 population.

Table S5. Premature mortality, YLL rates and age-adjusted YLL rates from COVID-19 for adults (>20 years) in Brazil

Age group	Population	Crude YLL weight*	Absolute YLL**	YLL rate†	Std. Population weigh	Age-standardized YLL
20-29	34,176,190	0.0430	67,583	197.7	0.1615	31.9
30-39	34,847,433.5	0.1120	176,030	505.1	0.1476	74.6
40-49	29,769,049.5	0.1900	298,622.4	1,003.1	0.1263	126.7
50-59	24,399,579	0.2190	344,201.6	1,410.7	0.0992	139.9
60-69	17,167,107	0.2240	352,060.1	2,050.8	0.0668	137
70-79	8,912,350	0.1440	226,324.3	2,539.4	0.0373	94.7
> 80	3,380,032	0.0680	106,875.4	3,162	0.0154	48.7
Total	152,651,741	-	1,571,696.9	10,868.9	-	653.5

YLL: years of life lost; Std.: standard
*Distribution of absolute YLL across age groups estimated from SUS datasets.[8]
**We assume that the cumulative absolute YLL exhibit a distribution of 98.5% for adults over 20 years old[4].
†Expressed per 100.000 population.

Table S6. Premature mortality, YLL rates and age-adjusted YLL rates from COVID-19 for adults (>20 years) in Chile

Age group	Population	Crude YLL weight*	Absolute YLL**	YLL rate†	Std. Population weigh	Age-standardized YLL
20-29	3,069,281.5	0.0290	36,945.1	1,203.7	0.1615	194.4
30-39	3,017,506.5	0.0849	108,191.1	3,585.4	0.1476	529,2
40-49	2,633,478.5	0.1871	238,370.1	9,051.5	0.1263	1,143.2
50-59	2,366,950.5	0.2710	345,198	14,584.1	0.0992	1,446.7

60-69	1,789,192.5	0.2475	315,318.6	17,623.5	0.0668	1,177.3
70-79	1,013,944.5	0.1283	163,502.1	16,125.3	0.0373	601.5
> 80	571,326.5	0.0387	49,287.3	8,626.8	0.0154	132.9
Total	14,461,68.5	-	1,256,812.3	70,800.4	-	5,225.1

YLL: years of life lost; Std.: standard
*Distribution of absolute YLL across age groups estimated from Salinas-Escudero et al.[4]
**We assume that the cumulative absolute YLL exhibit a distribution of 98.5% for adults over 20 years old[4].
†Expressed per 100.000 population.

Table S7. Premature mortality, YLL rates and age-adjusted YLL rates from COVID-19 for adults (>20 years) in Colombia

Age group	Population	Crude YLL weight*	Absolute YLL**	YLL rate†	Std. Population weigh	Age-standardized YLL
20-29	8,921,253	0.0290	50,720.5	568.5	0.1615	91.8
30-39	8,035,574	0.0849	148,531.4	1,848.4	0.1476	272.8
40-49	6,476,015.5	0.1871	327,249.3	5,053.3	0.1263	638.2
50-59	5,669,083	0.2710	473,909.2	8,359.5	0.0992	829.3
60-69	3,845,141	0.2475	432,888.9	11,258.1	0.0668	752
70-79	1,930,902	0.1283	224,465.7	11,624.9	0.0373	433.6
> 80	729,633.5	0.0387	67,664.7	9,273.8	0.0154	142.8
Total	35,607,602	-	1,725,429.8	47,986.5	-	3.160.6

YLL: years of life lost; Std.: standard
*Distribution of absolute YLL across age groups estimated from Salinas-Escudero et al.[4]
**We assume that the cumulative absolute YLL exhibit a distribution of 98.5% for adults over 20 years old[4].
†Expressed per 100.000 population.

Table S8. Premature mortality, YLL rates and age-adjusted YLL rates from COVID-19 for adults (>20 years) in México

Age group	Population	Absolute YLL	YLL rate*	Std. Population weigh	Age-standardized YLL
20-29	21,479,518	61,658	287.1	0.1615	46.4
30-39	18,897,970	180,561	955.5	0.1476	141
40-49	16,198,907	397,818	2,455.8	0.1263	310.2
50-59	12,720,337	576,104	4,529	0.0992	449.3
60-69	8,199,671	526,238	6,417.8	0.0668	428.7
70-79	4,225,668	272,870	6,457.4	0.0373	240.9
> 80	2,035,415	82,256	4,041.2	0.0154	62.2
Total	83,757,486	2,097,505	25,143.8	-	1,678.6

YLL: years of life lost; Std.: standard
*Expressed per 100.000 population.

Table S9. Premature mortality, YLL rates and age-adjusted YLL rates from COVID-19 for adults (>20 years) in Perú

Age group	Population	Crude YLL weight*	Absolute YLL**	YLL rate†	Std. Population weigh	Age-standard-ized YLL
20-29	5,496,385	0.0200	45,767.9	832.7	0.1615	134.5
30-39	4,980,552.5	0.0790	180,783.2	3,629.8	0.1476	535.8
40-49	4,106,757.5	0.1600	366,143.3	8,915.6	0.1263	1,126
50-59	3,063,742	0.2490	569,810.5	18,598.5	0.0992	1,845
60-69	2,151,175	0.2760	631,597.2	29,360.6	0.0668	1,961.3
70-79	1,239,255.5	0.1730	395.892,4	31.946,0	0.0373	1,191.6
> 80	547,489	0.0430	98.401,0	17,973.1	0.0154	276.8
Total	21.585,356.5	-	2,288,395.5	111256.3	-	

YLL: years of life lost; Std.: standard
*Distribution of absolute YLL across age groups estimated from peruvian ministry of health [13]
**We assume that the cumulative absolute YLL exhibit a distribution of 98.5% for adults over 20 years old[4].
†Expressed per 100.000 population.

4. Excess mortality estimation

We identified from the systematic review and prioritized published papers that accomplish the following *a priori* selection criteria: longer pre-vaccine analysis period, report COVID-19 mortality during the analysis period, and data stratification by sex and age. All the gathered data from country-specific studies have been cross-referenced and validated against the findings of the World Health Organization (WHO) study.[20]

When the excess mortality in individuals under 18 years of age was less than 10% compared to adults, the overall excess mortality for all age groups was reported. While estimation methods may vary across studies, this criterion ensures a more consistent description of the results

We extract from these papers the observed deaths (distinguishing between deaths resulting from natural causes and those attributed to COVID-19), total excess deaths (excess deaths due to natural causes plus deaths due to COVID-19), proportion of excess deaths due to COVID-19 ([Deaths due to COVID-19 / Total excess deaths] * 100); P-score ([Observed deaths - Expected deaths) / Expected deaths] * 100).

Table S10. characteristics of studies reporting overall excess deaths in six LATAM countries.

Study	Country	Oberved death (period)	Expected death (period)	Data source	Expected deaths (n)	Oberved deaths (n)	COVID-19 deaths (n)	Total excess deaths (n)	P-score
Rearte A et al. (2021)[6]	Argentina	2020	2015-19	DEIS y Registro Civil	342,341	378,647	45,568	36,306	10.6
dos Santos AM et al. (2021)[7]	Brazil	2020	2015-19	Ministério da Saúde	1,364,603	1,551,673	208,518	187,070	13.7
Msemburi W et al. (2020)[20]	Chile	2020	2015-19	WHO	NR	NR	16,499	14,575	13.1
Msemburi W et al. (2020)[20]	Colombia	2020	2015-19	WHO	NR	NR	42,620	54,060	21.8
Palacio-Mejía LS et al. (2021)[10]	Mexico	2020	2015-19	INEGI, RENAPO	723,773	1,050,383	125,485	326,610	45.1
Huarcaya WV et al (2022)[11]	Peru	Jan 2020 to Jun 2021	2017-19	SINADEF-INEI	166,519	349,756	100,955	183,237	109.5

DEIS: Dirección de estadísticas e información de la Salud; DANE: Departamento administrativo nacional de estadística; INEGI: Instituto nacional de Estadíistica y Geografía; RENAPO: Registro nacional de población; INEI: Instituto nacional de Estadística e Informática; SINADEF: sistema informático nacional de defunciones; M: male; F: female; N/R: not reported

Table S11. Excess deaths in six LATAM countries by sex.

Study	Country	Male expected deaths (n)	Female expected deaths (n)	Male observed deaths (n)	Female observed deaths (n)	Male COVID-19 deaths (n)	Female COVID-19 deaths (n)	Male total excess	Female total excess
Rearte A et al. (2021)[6]	Argentina	N/R	N/R	N/R	N/R	N/R	N/R	N/R	N/R
Santos AMD et al. (2021)[7]	Brazil	752,451	611,671	870,431	680,593	119,309	89,197	117,980	89,197
Msemburi W et al. (2020)[20]	Chile	N/R	N/R	N/R	N/R	N/R	N/R	N/R	N/R
Msemburi W et al. (2020)[20]	Chile	N/R	N/R	N/R	N/R	N/R	N/R	N/R	N/R
Palacio-Mejía LS et al. (2021)[10]	México	408,519	315,254	617,921	432,462	73,707†	51,778†	209,402	117,208
Huarcaya WV et al (2022)[11]	Perú	NR	NR	NR	NR	N/R	N/R	NR	NR

N/R: not reported
*Data are expressed as excess mortality for all ages. Observed and Expected values were estimated using p-score derived from excess mortality between 15 and 69 years.
† Data was estimated using male and female fatality rate (Mexican MOH database) and total deaths from Palacio-Mejía et al.

5. Length of stay in hospitalization

Four regional studies were identified that provided data on length of hospitalization in Chile, Colombia, Mexico, and one that covered several countries in Latin America and the Caribbean.[15,21–23] Regarding Brazil, information on days of hospitalization according to severity was obtained through the Ministerial database. Based on these findings, the days of hospitalization were approximated according to severity and type of ward. A process of validation, adaptation and completeness of this data was carried out according to the specific needs of this study, using the information presented in the LATAM study and the data obtained from the Brazilian database. The objective was to ensure that the days of hospitalization in the ICU or ICU with mechanical ventilation in severe patients were within the range presented by the LATAM study in all countries, and in critical patients were close to the data provided by the information from Brazil. Table S12 presents the days of hospitalization in the general ward, ICU and ICU with mechanical ventilation by severity of COVID and for each of the countries of the analysis.

Table S12. Length of stay in days

	Argentina	Brasil	Chile	Colombia	Mexico	Peru
**Moderate and severe COVID-19 case**						
General ward	6.3	8.3	9.9	6.3	6.3	9
ICU without ventilatory support	2.1	1.7	2.1	2.4	2.1	1.8
**Critical COVID-19 case**						
General ward	2.0	9.3	6.6	4.2	2.1	6.0
ICU without ventilatory support	4.2	1.6	3.0	1.6	3.0	1.2
ICU with ventilatory support	15.4	10.7	18.9	7.2	9.9	14.5

Source: own elaboration

Table S13 shows the specific resource utilization for each COVID severity level, which was used in the calculation of costs by severity level.

Table S13. Use of resources by severity level

Resource	Mild COVID-19	Moderate and severe COVID-19 case	Critical COVID-19
Emergency consultation	0.05	0.80	0.90
General medicine consultation	0.95	0.20	0.10
COVID-19 diagnosis	1.00	1.00	1.00
Follow-up consultations	1.60	NA	NA
General ward	NA	6.30	2.04
ICU without ventilatory support	NA	2.10	4.22
ICU with ventilatory support	NA	NA	15.39
Blood culture	NA	1.00	1.00
Blood count	NA	3.60	15.00
Glycemia	NA	3.60	15.00
Creatinine	NA	3.60	15.00
Hepatogram	NA	1.80	7.00
Ionogram	NA	3.60	15.00
Blood gases	NA	6.30	30.00
Electrocardiogram	NA	0.90	3.00
Chest x-ray	NA	2.70	15.00

Thorax tomography	NA	0.45	0.80
Chest ultrasound	NA	NA	5.00
Echocardiogram	NA	NA	5.00
Ibuprofen in milligram	4,320.00	7,200.00	NA
Propofol in milligram	NA	NA	9,408.00
Midazolam in milligram	NA	NA	2,661.12
Dexmedetomidine in microgram	NA	NA	4,116.00
Fentanile in microgram	NA	NA	96,768.00
Remifentanil in milligram	NA	NA	28.22
Atracurium in milligram	NA	NA	51.74
Rocuronium in milligram	NA	NA	0.84
Vecuronium in milligram	NA	NA	5.04
Dexamethasone in milligram	NA	NA	64.00
Enoxaparin in milligram	NA	NA	504.00
Heparin in international unit	NA	NA	94,500.00
Tocilizumab in milligram	NA	NA	5.00

Source: own elaboration

6. Unit resource costs by severity of COVID-19

To determine unit costs, a search of cost databases was carried out in the countries subject to analysis, where available data for Argentina, Brazil, Chile and Colombia were identified. Both the nomenclators and tariff manuals of the public and private system were considered in those countries where this information was available. In Argentina, the healthcare system operates across three sectors: public, social security, and private. The social security sector, covering about 46% of the Argentine population, is the largest, followed by the public sector covering approximately 36%, and the private sector at 18%. For our analysis, a weighted average was carried out by coverage. In Brazil, the healthcare system is centralized under a single system known as the Unified Health System (SUS). For our calculations, we utilized values from the official SUS nomenclator, the Sistema de Gerenciamento da Tabela de Procedimentos, Medicamentos e OPM do SUS (SIGTAP). These values were inflation-adjusted starting from 2017, assuming that the values presented in the SIGTAP reflect those corresponding to the year of its publication, as mentioned in the technical note Competência - 03/2017. In the case of Colombia, we referenced the rate manual of the Social Security Institute (ISS2001), adjusting 30% upwards and inflating from 2010 to August 2023, following the methodological manual by IETS. Additionally, we factored in the value of Mandatory Traffic Accident Insurance (Seguro Obligatorio contra Accidentes de Tránsito - SOAT-) for 2023. We calculated an average between ISS2001 and SOAT values to obtain a final figure. In the case of Chile, we considered both the National Health Fund (Fondo Nacional de Salud -FONASA-) and the Pension Health Institutions (Instituciones de Salud Previsional -ISAPRE-), taking into account the respective available nomenclators for these subsystems. The FONASA values were adjusted for inflation from 2022, while those from ISAPRE were adjusted from 2019 to August 2023. Subsequently, using Gattini's data on coverage, data on coverage, we calculated a weighted average by affiliation to each subsystem (FONASA: 79.2% and ISAPRES: 20.8%).

The drugs were selected based on the severity of COVID-19. Only non-steroidal anti-inflammatory Drugs (NSAIDs) were included in mild and moderate/severe cases, while in critical cases, sedatives, analgesics, and neuromuscular blockers were included. In addition to the medications described in severe cases, anticoagulants were also included in critical cases. The doses of each drug were calculated according to what is recommended for an adult weighing 70 kilos.

Table S14 shows the unit costs of each resource used in the analysis for each country according to the severity of COVID-19. All values are expressed in US dollars, using the average data for August provided by the central bank of each country.

Table S14. Unit costs of each resource used according to the severity of COVID-19

Resource	Argentina	Brasil	Chile	Colombia	Mexico*	Peru*
Exchange rate national monetary unit to dollar	ARS$350.00	BRL$4.90	CLP$855.66	COP$4,066.89	MXN$16.98	PEN$3.69
**Mild COVID-19 case**						
Emergency consultation	$9.4159	$2.7730	$25.9992	$7.5534	$10.6236	$6.8253
General medicine consultation	$7.6554	$2.7730	$17.7077	$5.3223	$7.8366	$5.0348
COVID-19 diagnosis	$45.4914	$17.5995	$9.5689	$13.0852	$21.8892	$14.0631
Follow-up consultations	$7.6554	$2.7730	$17.7077	$5.3223	$7.8366	$5.0348
Ibuprofen per milligram	$0.0008	$0.0004	$0.0001	$0.0004	$0.0004	$0.0003
**Moderate and severe COVID-19 case**						
Emergency consultation	$9.4159	$2.7730	$25.9992	$13.1450	$12.9620	$8.3277
General medicine consultation	$7.6554	$2.7730	$17.7077	$8.3658	$9.1094	$5.8525
COVID-19 diagnosis	$45.4914	$17.5995	$9.5689	$13.0852	$21.8892	$14.0631
General ward	$228.4777	$40.7629	$194.4623	$46.8244	$113.6622	$73.0243
ICU without ventilatory support	$387.4199	$371.5804	$353.2826	$401.3480	$425.6697	$273.4790
Blood culture	$11.3729	$3.1862	$27.0546	$20.5020	$16.7543	$10.7641
Blood count	$3.4119	$1.1369	$4.8462	$7.3052	$4.9749	$3.1962
Glycemia	$1.7059	$1.8163	$1.9950	$3.3136	$2.6564	$1.7066
Creatinine	$3.9805	$1.0260	$2.0498	$3.4791	$2.9506	$1.8957
Hepatogram	$6.8237	$2.6454	$15.8677	$17.4807	$12.3809	$7.9543
Ionogram	$3.9803	$2.0520	$2.3794	$20.3101	$10.3679	$6.6611
Blood gases	$11.3729	$0.7709	$7.6090	$20.1834	$12.3587	$7.9400
Electrocardiogram	$8.0330	$1.4281	$9.1964	$11.9391	$8.7252	$5.6057
Chest x-ray	$9.5460	$2.4208	$17.6128	$35.0856	$20.5402	$13.1964
Thorax tomography	$47.5501	$37.8263	$155.3061	$59.4432	$74.2992	$47.7348
Ibuprofen per milligram	$0.0008	$0.0004	$0.0001	$0.0004	$0.0005	$0.0003
**Critical COVID-19 case**						
Emergency consultation	$9.4159	$2.7730	$25.9992	$13.1450	$12.9620	$8.3277
General medicine consultation	$7.6554	$2.7730	$17.7077	$8.3658	$9.1094	$5.8525
COVID-19 diagnosis	$45.4914	$17.5995	$9.5689	$13.0852	$21.8892	$14.0631
General ward	$228.4777	$40.7629	$194.4623	$46.8244	$113.6622	$73.0243
ICU without ventilatory support	$387.4199	$371.5804	$353.2826	$401.3480	$425.6697	$273.4790
ICU with ventilatory support	$423.0514	$394.8735	$363.4116	$412.2036	$446.5003	$286.8620
Blood culture	$11.3729	$3.1862	$27.0546	$20.5020	$16.7543	$10.7641
Blood count	$3.4119	$1.1369	$4.8462	$7.3052	$4.9749	$3.1962
Glycemia	$1.7059	$1.8163	$1.9950	$3.3136	$2.6564	$1.7066
Creatinine	$3.9805	$1.0260	$2.0498	$3.4791	$2.9506	$1.8957
Hepatogram	$6.8237	$2.6454	$15.8677	$17.4807	$12.3809	$7.9543
Ionogram	$3.9803	$2.0520	$2.1824	$20.3101	$10.3323	$6.6382
Blood gases	$11.3729	$0.7709	$7.6090	$20.1834	$12.3587	$7.9400
Electrocardiogram	$8.0330	$1.4281	$9.1964	$11.9391	$8.7252	$5.6057
Chest x-ray	$9.5460	$2.4208	$17.6128	$35.0856	$20.5402	$13.1964
Thorax tomography	$47.5501	$37.8263	$155.3061	$59.4432	$74.2992	$47.7348
Chest ultrasound	$36.9179	$6.7106	$26.2959	$24.4591	$24.5435	$15.7684
Echocardiogram	$35.1612	$18.8175	$112.9176	$54.2523	$56.0535	$36.0125
Propofol per milligram	$0.6057	$0.6239	$0.4952	$0.0085	$0.4097	$0.1125
Midazolam per milligram	$0.5729	$1.8475	$0.4333	$0.2373	$0.8680	$0.0399

Dexmedetomidine per microgram	$0.2276	$0.3481	$0.1375	$0.0049	$0.1812	$0.0676
Fentanile per microgram	$0.0424	$0.0108	$0.0186	$0.0011	$0.0158	$0.0027
Remifentanil per milligram	$10.2603	$6.5986	$16.6608	$3.1425	$8.4532	$7.0984
Atracurium per milligram	$0.5938	$0.6393	$0.0997	$0.0717	$0.3671	$12.20
Rocuronium per milligram	$0.3321	$0.0939	$0.1830	$0.0359	$0.1445	$0.04
Vecuronium per milligram	$3.9865	$3.0641	$2.1162	$0.4186	$2.3176	$2.12
Dexamethasone per milligram	$1.4489	$0.0108	$0.6410	$0.1126	$0.4598	$1.08
Enoxaparin per milligram	$0.7007	$0.0020	$0.4475	$0.1147	$0.4230	$68.01
Heparin per international unit	$0.0051	$0.0022	$0.0001	$0.0005	$0.1429	$102.01
Tocilizumab per milligram	$4.3663	$21.9091	$1.8882	$0.9627	$7.5569	$11.98

*Indirect estimation

Source: own elaboration

7. Other vaccine-preventable diseases

For data regarding incidence and deaths of Pneumonia and Influenza we explored ministerial reports.

Table S15. Sources searched for country-specific epidemiological data.

Country	Data source	Link
Argentina	MOH; DEIS<br>Dirección de Control de Enf. Inmunoprevenibles	https://www.argentina.gob.ar/salud/deis; http://www.datos.salud.gob.ar/<br>https://www.argentina.gob.ar/salud/inmunoprevenibles
Brazil	MOH	https://www.gov.br/saude/pt-br/composicao/svsa
Chile	MOH; DEIS	https://www.minsal.cl/; https://deis.minsal.cl/
Colombia	MOH; DANE	https://www.datos.gov.co/; https://www.dane.gov.co/
México	RENAPO, INEGI	https://www.gob.mx/segob/renapo; https://www.inegi.org.mx/
Perú	MOH; INEI	https://www.minsa.gob.pe/defunciones/; https://www.gob.pe/inei/
All Countries	PAHO<br>Our World in Data	https://www.paho.org/es/sireva<br>https://ourworldindata.org/grapher/pneumonia-and-lower-respiratory-diseases-deaths

MOH: Ministry of Health; DEIS: Directorate of Health Statistics and Information; DANE: National Administrative Department of Statistics; RENAPO: General Directorate of the National Registry of Population and Identity; INEGI: National Institute of Statistic and Geography; INEI: National Institute of Statistics and Informatics.

The periods selected according to the vaccination programme for each country were: Influenza:

Argentina	Jan 2001 to Dec 2003
Brasil	Jan 2004 to Dec 2006
Chile	Jan 2005 to Dec 2007

Colombia	Jan 2002 to Dec 2004
Mexico	Jan 2002 to Dec 2004
Peru	Jan 2002 to Dec 2004

Pneumococcus:

Argentina	Jan 2015 to Dec 2017
Brasil	Jan 2008 to Dec 2010
Chile	Jan 2008 to Dec 2010
Colombia	Jan 2009 to Dec 2011
Mexico	Jan 2004 to Dec 2006
Peru	Jan 2007 to Dec 2009

Table S16. Pneumonia and Influenza average death counts, by country and age.

	Argentina	Brazil	Chile	Colombia	Mexico	Peru
Death counts all	36808	56081	3737	5415	12672	14554

Note: Deaths from 'clinical pneumonia', which refers to a diagnosis based on disease symptoms such as coughing and difficulty breathing and may include other lower respiratory diseases. Source: IHME, Global Burden of Disease (2019). Average cases over a three year period

8. Search strategy for information on costs of immune preventable diseases

For cost data extraction, a literature search was performed using the PubMed and Lilacs databases to estimate costs associated with pneumococcal disease and influenza. Studies of economic burden or direct medical costs were identified for each vaccine-preventable disease in Argentina, Brazil, Chile, Colombia and Peru; no economic studies were found that met inclusion criteria for Mexico. Seven studies on pneumococcus and three on influenza were included. In the particular case of pneumococcal disease, since not all studies had the same definition of pneumococcal disease, it was decided to take all invasive diseases defined as consolidated pneumonia, focal pneumonia, and bacteremia/sepsis as pneumococcal disease for our cost extraction. From each study, the number of cases reported for each type of pneumococcal disease was taken and a weighted average was made, so that we could obtain a value per hospitalized case of pneumococcal disease. The costs were updated for inflation as of June 2023 . Official data from each country were used to adjust for inflation [24–28]. Costs are reported in US dollars for the year 2023. The search strategies used, the databases, and the results obtained from them are detailed below.

Pubmed

Pneumococcal

(Pneumococcal Infections[Mesh] OR Pneumococc*[tiab] OR Streptococc*[tiab]) AND (Value of Life[Mesh] OR Economics, Dental[Mesh] OR Economics, Hospital[Mesh] OR Economics, Medical[Mesh] OR Economics, Pharmaceutical[Mesh] OR Economics, Nursing[Mesh] OR "Fees and Charges"[Mesh] OR Budgets[Mesh] OR Models, Economic[Mesh] OR "Costs and Cost Analysis"[Mesh] OR Cost[tiab] OR Costs[tiab] OR Costed[tiab] OR Costly[tiab] OR Economic*[ti] OR Pharmacoeconomic*[tiab] OR Price*[tiab] OR Pricing[tiab] OR Contingent Valuat*[tiab] OR "Willingness to Pay"[tiab] OR Conjoint Analysis[tiab] OR DALY*[tiab] OR QALY*[tiab] OR Burden[tiab] OR Quality-Adjusted[tiab] OR Expenditure*[tiab] OR Out-of-Pocket[tiab] OR Health Resources[Mesh]) AND (Argentina[Mesh] OR Argentin*[tiab] OR Brazil[Mesh] OR Brazil*[tiab] OR Chile[Mesh] OR Chile*[tiab] OR Colombia[Mesh] OR Colombia*[tiab] OR Mexico[Mesh] OR Mexic*[tiab] OR Peru[Mesh] OR Peru*[tiab])

Results: 146 articles
Excluded by title and abstract: 139
For full review: 7

Influenza

(Influenza, Human[Mesh] OR Influenza[tiab] OR Flu[tiab] OR Grippe[tiab]) AND (Value of Life[Mesh] OR Economics, Dental[Mesh] OR Economics, Hospital[Mesh] OR Economics, Medical[Mesh] OR Economics, Pharmaceutical[Mesh] OR Economics, Nursing[Mesh] OR "Fees and Charges"[Mesh] OR Budgets[Mesh] OR Models, Economic[Mesh] OR "Costs and Cost Analysis"[Mesh] OR Cost[tiab] OR Costs[tiab] OR Costed[tiab] OR Costly[tiab] OR Economic*[ti] OR Pharmacoeconomic*[tiab] OR Price*[tiab] OR Pricing[tiab] OR Contingent Valuat*[tiab] OR "Willingness to Pay"[tiab] OR Conjoint Analysis[tiab] OR DALY*[tiab] OR QALY*[tiab] OR Burden[tiab] OR Quality-Adjusted[tiab] OR Expenditure*[tiab] OR Out-of-Pocket[tiab] OR Health Resources[Mesh]) AND (Argentina[Mesh] OR Argentin*[tiab] OR Brazil[Mesh] OR Brazil*[tiab] OR Chile[Mesh] OR Chile*[tiab] OR Colombia[Mesh] OR Colombia*[tiab] OR Mexico[Mesh] OR Mexic*[tiab] OR Peru[Mesh] OR Peru*[tiab])

Results: 193 articles
Excluded by title and abstract: 190
For full review: 3

Lilacs

(MH Pneumococcal Infections OR Pneumococ$ OR Neumococ$ OR Streptococ$ OR Estreptococ$ OR MH Influenza, Human OR Influenza OR Flu OR Grippe OR Gripe) AND (MH Value of Life OR MH Economics, Dental OR MH Economics, Hospital OR MH Economics, Medical OR MH Economics, Pharmaceutical OR MH Economics, Nursing OR MH Budgets OR MH Models, Economic OR MH Costs and Cost Analysis OR Cost OR Costs OR Costed OR Costo OR Costos OR Custo OR Costly OR TI Economic$ OR Pharmacoeconomic$ OR Farmacoeconom$ OR Price$ OR Precio$ OR Preço$ OR Pricing OR Contingent$ OR ((Willingness OR Voluntad OR Vontade OR Disposición) AND (Pay OR Pago OR Pagar OR Conjoint OR DALY$ OR QALY$ OR Burden OR Carga OR Quality-Adjusted OR Expenditure$ OR Gasto$ OR Despesa$ OR Out-of-Pocket OR MH Health Resources) AND (MH Argentina OR Argentin$ OR MH Chile OR Chil$ OR MH Colombia OR Colombi$ OR MH Mexico] OR Mexic$ OR MH Peru] OR Per$)

Results: 35 articles
Excluded by title and abstract: 35
For full review: 0

Table S17 presents the results of the costs of other immune preventable diseases collected from each study, updated to June 2023 and expressed in USD.

Table S17. Costs per hospitalized case of pneumococcal and influenza in USD 2023

Author, year	Country	Average cost per hospitalization
**Pneumococcus**		
Giglio et al, 2022[29]	Argentina	$1.413,06
Michelin et al, 2017[30]	Brasil	$1.426,50
Andrade et al, 2017[31]		$838,57
Biagini et al, 2018[32]	Chile	$1.464,24
Castañeda et al, 2011[33]		$899,26
Ordóñez et al, 2023[34]	Colombia	$1.501,48
Pugh et al, 2020[35]		$1.930,54
**Influenza**		
Ureña 2021[36]	Argentina	$391,88
Tinoco 2015[37]	Peru	$21,73
Hung Nguyen 2020[38]	Argentina	$97,96

Source: own elaboration

9. Population data by country

Table S18. Population data from 2020 by country, sex and age

Indicators	Argentina	Brazil	Chile	Colombia	Mexico	Perú
Population both sex	32,299,133	159,259,824	14,981,552	37,315,877	87,519,061	22,698,846
18-49 yrs	20,686,880	105,400,756	9,240,138	25,141,118	60,118,789	15,697,185
50-64 yrs	6,330,671	34,051,521	3,349,028	7,859,755	17,293,650	4,253,175
>=65yrs	5,281,580	19,807,546	2,392,385	4,315,004	10,106,622	2,748,486
Female	16,542,390	81,973,388	7,602,222	19,118,939	45,445,190	11,586,869
18-49 yrs	10,186,283	52,766,454	4,574,706	12,620,619	30,809,938	7,952,976
50-64 yrs	3,239,481	17,892,704	1,703,701	4,101,277	9,139,462	2,162,104
>=65yrs	3,116,626	11,314,305	1,323,815	2,397,042	5,495,790	1,471,788
Male	15,756,742	77,286,436	7,379,329	18,196,938	42,073,871	11,111,977
18-49 yrs	10,500,599	52,634,302	4,665,432	12,520,499	29,308,851	7,744,208
50-64 yrs	3,091,189	16,158,817	1,645,327	3,758,478	8,154,188	2,091,071
>=65yrs	2,164,954	8,493,316	1,068,570	1,252,049	4,610,831	1,276,698

Note: Data from United Nation 2020 (https://www.un.org/development/desa/pd/data-landing-page)

Table S19A. COVID-19 cases under critical care counts and percentage by country, age, and sex.

Indicators	Argentina	Brazil	Chile *	Colombia *	Mexico	Peru
Critical care both sex n (%)	33,834 (100)	478,171 (100)	18,537 (100)	33,503 (100)	37,113 (100)	11,299 (100)
18-49 yrs	5,535 (16)	115,128 (24)	1,044 (6)	5,481 (16)	9,822 (2)	3,853 (34)
50-64 yrs	10,453 (30)	150,477 (31)	5,689 (31)	10,351 (31)	13,597 (37)	3,923 (35)
>=65 yrs	17,846 (54)	212,566 (45)	11,804 (63)	17,671 (53)	13,691 (37)	3,523 (31)
Critical care Female	12,615 (37)	204,590 (43)	7,377 (40)	11,664 (35)	13,567 (37)	3,689 (33)
18-49 yrs	2,092 (17)	44,462 (22)	415 (5)	1,938 (16)	3,370 (25)	1,397 (38)
50-64 yrs	3,473 (27)	61,671 (30)	2,264 (31)	3,209 (28)	4,821 (35)	1,067 (29)
>=65 yrs	7,050 (56)	98,457 (48)	4,698 (64)	6,517 (56)	5,376 (40)	1,225 (33)
Critical care Male	20,691 (61)	273,547 (57)	11,159 (60)	21,839 (65)	23,543 (63)	7,578 (67)
18-49 yrs	3,383 (16)	70,656 (26)	628 (5)	3,580 (16)	6,452 (27)	2,427 (32)
50-64 yrs	6,901 (33)	88,798 (32)	3,425 (31)	7,288 (34)	8,776 (37)	2,853 (38)
>=65 yrs	10,407 (51)	114,093 (42)	7,106 (64)	10,971 (50)	8,315 (36)	2,298 (30)

Notes: Missings: Argentina (0.9-2.2%), Brazil (0.01 %) Chile, Colombia, Mexico and Peru (0%). Critical care refers to the need of hospitalization in intensive care units. *Values estimated from published studies(7)(from February 2020 until 1st February 2021 for Chile (12 months), until 1st June 2021 for Argentina, Brazil, Colombia, Mexico and Peru (16 months).

Table S19B. COVID-19 Hospitalized patients who required mechanical ventilation, counts, and percentage by country, sex, and age.

Indicators	Argentina	Brazil	Chile*	Colombia*	Mexico	Peru
COVID-19 Hospitalization n	167,173	1,314,569	53,773	103,402	464,804	98,095
Hospitalized cases with mechanical ventilation (%)	12	20	16	18	14	9
Mechanical Ventilation both sex n (%)	19,387 (100)	268,411 (100)	8,386 (100)	18,174 (100)	62,640 (100)	8,576 (100)
18-49 yrs	2,998 (15)	55,780 (21)	1,677 (20)	2,810 (15)	13,440 (22)	2,921 (34)
50-64 yrs	6,544 (34)	85,193 (32)	4,696 (56)	6,135 (34)	23,831 (38)	3,138 (37)
>=65 yrs	9,845 (51)	127,438 (47)	2,013 (24)	9,229 (51)	25,369 (40)	2,517 (29)
Mechanical Ventilation Female	6,809 (35)	116,458 (43)	4,864 (58)	4,907 (27)	22,463 (36)	2,595 (30)
18-49 yrs	1,089 (16)	22,578 (19)	972 (20)	780 (16)	4,240 (19)	935 (36)
50-64 yrs	2,116 (31)	35,830 (31)	2,724 (56)	1,526 (31)	8,415 (37)	830 (32)
>=65 yrs	3,604 (53)	58,050 (50)	1,167 (24)	2,601 (53)	9,808 (44)	830 (32)
Mechanical Ventilation Male	12,320 (64)	151,932 (57)	3,522 (42)	13,267 (73)	40,177 (64)	5,953 (70)
18-49 yrs	1,875 (15)	33,195 (22)	704 (20)	2,016 (15)	9,200 (23)	1,961 (33)
50-64 yrs	4,380 (36)	49,358 (32)	1,972 (56)	4,723 (36)	15,416 (38)	2,305 (39)
>=65 yrs	6,065 (49)	69,379 (46)	845 (24)	6,527 (49)	15,561 (39)	1,687 (28)

Notes:**Missings: Argentina (0.9%-.8%), Brazil (0.01 %), Peru (0.3-0.5%) Chile, Colombia and Mexico (0%) * Values estimated from published studies (7)(from February 2020 until 1st February 2021 for Chile (12 months), until 1st June 2021 for Argentina, Brazil, Colombia, Mexico, and Peru (16 months)).

Table S20. Comorbidities in COVID-19 cases by country.

Country	Argentina	Brazil**	Chile	Colombia ***	Mexico	Perú
Source and period	SIISA database[17] (Mar 2020 to Oct 2020)	MOH (Jan 2020 to Jun 2021)	MOH (Jan 2020 to Jan 2021)	[39]	MOH (Jan 2020 to Jun 2021)	Soto-Cabezas et al[40] (Mar 2020 to oct 2020))
COVID-19, N	207,079	1,496,440	728,812	3,03	2,363,214	51,503
Asthma, n (%)	12,580 (6.1)	35,017 (2.3)	23,668 (3.2)	100 (3.3)	50,343 (2.1)	637 (1.2)
Chronic neurologic disease, n (%)	5,356 (2.6)	40,255 (3.1)	3,813 (0.5)	NA	NA	536 (1)
COPD, n (%)	4,405 (2.1)	45,287 (3)****	7,508 (1)****	168 (5.5)	26,711 (1.1)	847 (1.6)
CKD, n (%)	2,324 (1.1)	48,060 (3.2)	6,173 (0.8)	235 (7.8)	35,943 (1.5)	1,144 (2.2)
CVD, n (%)	NA	482,361 (32.2)	7,052 (1)	198 (6.5)	37,003 (1.6)	8,059 (8.6)
CHF, n (%)	5,753 (2.8)	NA	6,417 (1)	137 (4.5)	NA	NA
Diabetes, n (%)	20,058 (9.7)	346,818 (23.2)	54,949 (7.5)	619 (20.4)	321,581 (13.6)	5,737 (11.1)
Hypertension, n (%)	39,833 (19.2)	NA	105,077 (14.4)	1,237 (40.8)	418.,004 (17.7)	NA
Chronic liver disease, n (%)	914 (0.4)	10,966 (0.7)	1,241 (0.2)	NA	NA	279 (0.5)
ID, n (%)	2,972 (1.4)	30,162 (2)	4,471 (0.6)	NA	NA	157 (0.3)
Obesity, n (%)	10,854 (5.2)	117,850 (7.9)	23,343 (3.2)	658 (21.7)	343,763 (14.5)	2,649 (5.1)
Tobacco use, n (%)	9,530 (4.6)*****	NA	NA	NA	178,595 (7.6)	NA

Note: all % are related to the total number of COVID-19 patients for each country. NA: not available; COPD: chronic obstructive pulmonary disease; CKD: chronic kidney disease; CVD: cardiovascular disease; CHF: chronic heart failure; ID: Immunodeficiency IS: immunosuppression. *Representative sample of Autonomous city of Buenos Aires **Only severe and critical cases *** Patients reported to the emergency room from the designated hospital for COVID-19. ****Defined as chronic pulmonary disease. ***** Sum of current and former smokers.

Table S21. COVID-19 Death counts and percentages by country, sex and age.

Indicators n (%)	Argentina	Brazil	Chile	Colombia	Mexico	Peru
Death both sex	92,434 (100)	521,577 (100)	18,480 (100)	89, 137 (100)	237,947 (100)	184,969 (100)
18-49 yrs	6,816 (7)	88,035 (16.9)	1,041 (5.6)	8,928 (10)	37,678 (16)	23,947 (13)
50-64 yrs	19,257 (21)	140,505 (26.9)	5,679 (30.7)	22,104 (25)	82,553 (35)	54,055 (29)
>=65 yrs	66,361 (72)	293,037 (56.2)	11,760 (63.6)	58,105 (65)	117,716 (49)	106,967 (58)
Death Female	37,714 (41)	228,360 (43.8)	6,542 (35.4)	33,978 (38)	89, 116 (37)	66, 805 (36)
18-49 yrs	2,531 (7)	35,620 (15.6)	262 (4)	2,912 (9)	12,074 (14)	7,729 (12)
50-64 yrs	6,507 (17)	58,070 (25.4)	2,682 (40.9)	7,834 (23)	30,249 (34)	18,360 (27)
>=65 yrs	28,676 (76)	134,670 (58.9)	3,598 (54.9)	23,232 (68)	46,793 (52)	40,716 (61)
Death Male	52,728 (57)	293,159 (50)	11,938 (64.6)	55,159 (62)	148,831 (63)	118,164 (64)
18-49 yrs	4,201 (8)	52,402 (18)	477(4)	6,016 (11)	25,604 (17)	16,218 (14)
50-64 yrs	12,604 (24)	82,419 (28)	4,895 (41)	14,270 (26)	52,304 (35)	35,695 (30)
>=65 yrs	35,923 (68)	158,338 (54)	6,566 (55)	34,873 (63)	70,923 (48)	66,251 (56)
Mortality rate per 100000	276.1	327.5	123.4	238.8	271.8	814.9
Case fatality rate	2.5	4.4	2.5	2.8	10.1	9.9

Notes: Missings: Argentina (0.9- 2.7%), Brazil (both sex: 0.01-0.02%), Chile, Colombia , Mexico and Peru (0%). (from February 2020 until 1st February 2021 for Chile (12 months), until 1st June 2021 for Argentina, Brazil, Colombia, Mexico and Peru (16 months)).

Table S22. COVID-19 Case Fatality rate as percentage of deaths by country, sex and age.

Indicators	Argentina	Brazil	Chile	Colombia	Mexico	Peru
Death n	3,711,250	11,956,157	728,812	3,240,355	2,363,214	1,867,034
Fatality rate %	2.5	4.4	2.5	2.8	10.1	9.9
18-49 yrs	0.3	0.9	0.2	0.4	2.5	2.0
50-64 yrs	2.6	11.6	3.4	3.2	14.7	13.0
>=65 yrs	15.9	49.8	13.2	15.8	38.3	44.8
Fatality rate Female	2.0	3.9	1.8	0.4	7.5	7.4
18-49 yrs	0.2	0.7	0.1	0.3	1.6	1.3
50-64 yrs	1.8	10.1	3.3	2.2	10.9	9.3
>=65 yrs	13.5	47.3	7.6	12.1	32.9	35.7
Fatality rate Male	2.9	4.8	3.3	3.6	12.6	12.3
18-49 yrs	0.3	1.0	0.2	0.6	3.5	2.6
50-64 yrs	3.4	13.0	6.0	4.4	18.4	16.3
>=65 yrs	18.2	52.2	15.7	19.8	43.0	53.1

Figure S8. Monthly COVID-19 case fatality rate.

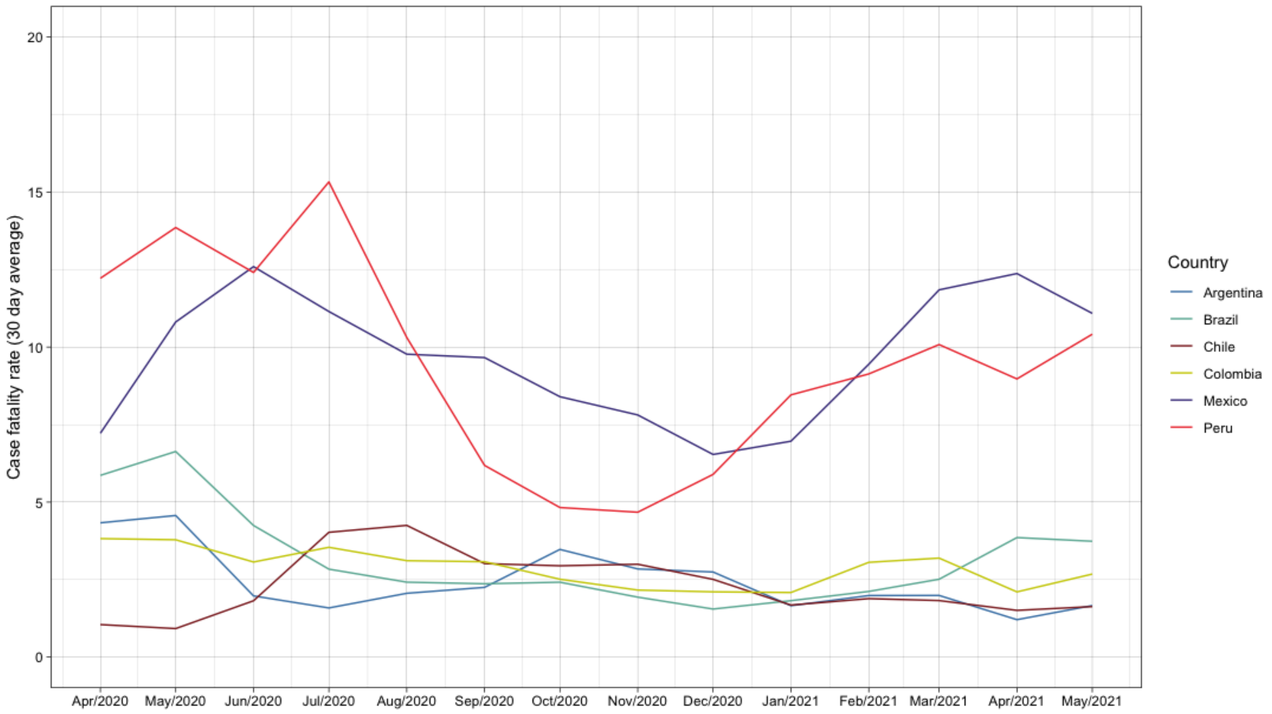

Note: This figure has been created and edited by the authors of this study using open-source data from Our World in Data.(28)

References

1. Pifarré I Arolas, H.; Acosta, E.; López-Casasnovas, G.; Lo, A.; Nicodemo, C.; Riffe, T.; Myrskylä, M. Years of Life Lost to COVID-19 in 81 Countries. *Sci Rep* **2021**, *11*, 3504.
2. de Castro, A.P.B.; Moreira, M.F.; de Souza Bermejo, P.H.; Rodrigues, W.; Prata, D.N. Mortality and Years of Potential Life Lost Due to COVID-19 in Brazil. *Int. J. Environ. Res. Public Health* **2021**, *18*, 1–17.
3. Islam, N.; Jdanov, D.A.; Shkolnikov, V.M.; Khunti, K.; Kawachi, I.; White, M.; Lewington, S.; Lacey, B. Effects of Covid-19 Pandemic on Life Expectancy and Premature Mortality in 2020: Time Series Analysis in 37 Countries. *BMJ* **2021**, *375*, e066768.
4. Ugarte, M.P.; Achilleos, S.; Quattrocchi, A.; Gabel, J.; Kolokotroni, O.; Constantinou, C.; Nicolaou, N.; Rodriguez-Llanes, J.M.; Huang, Q.; Verstiuk, O.; et al. Premature Mortality Attributable to COVID-19: Potential Years of Life Lost in 17 Countries around the World, January–August 2020. *BMC Public Health* **2022**, *22*, 1–13.
5. Salinas-Escudero, G.; Toledano-Toledano, F.; García-Peña, C.; Parra-Rodríguez, L.; Granados-García, V.; Carrillo-Vega, M.F. Disability-Adjusted Life Years for the COVID-19 Pandemic in the Mexican Population. *Front. Public Health* **2021**, *9*, 686700.
6. Rearte, A.; Moisés, M.S.; Rueda, D.V.; Laurora, M.A.; Marucco, A.F.; Pennini, V.A.; Giovacchini, C.M.; Guevel, C.; Vizzoti, C. All-cause excess mortality during the COVID-19 pandemic in Argentina, 2020. *Rev. argent. salud publica* **2021**, 18–18.
7. dos Santos, A.M.; de Souza, B.F.; de Carvalho, C.A.; Campos, M.A.G.; de Oliveira, B.L.C.A.; Diniz, E.M.; dos Remédios Freitas Carvalho Branco, M.; de Sousa Queiroz, R.C.; de Carvalho, V.A.; Araújo, W.R.M.; et al. Excess Deaths from All Causes and by COVID-19 in Brazil in 2020. *Rev. saúde pública* **2021**, *55*, 71–71.
8. Quapper, D.D. [Excess deaths due to the COVID-19 pandemic in Chile]. *Rev. Med Chil***.** **2021**, *149*, 1525–1531.
9. Benavides, F.G.; Vives, A.; Zimmerman, M.; Silva-Peñaherrera, M. [Excess Mortality in 2020 in the Working-Age Population of Nine Latin American countriesExcesso de Mortalidade Na População Em Idade Ativa Em Nove Países Da América Latina No Ano de 2020]. *Revista panamericana de salud publica = Pan American journal of public health* **2022**, *46*, doi:10.26633/RPSP.2022.75.
10. Palacio-Mejía, L.S.; Wheatley-Fernández, J.L.; Ordóñez-Hernández, I.; López-Ridaura, R.; Gatell-Ramírez, H.L.; Hernández-Ávila, M.; Hernández-Ávila, J.E. Estimación del exceso de mortalidad por todas las causas durante la pandemia del Covid-19 en México. *Salud pública Méx* **2021**, *63*, 211–224.
11. Huarcaya, W.V.; Monzón, J.A.M.; Saldaña, E.O.N.; Driver, C.R. Impacto de La COVID-19 En La Mortalidad En Perú Mediante La Triangulación de Múltiples Fuentes de Datos. *Revista Panamericana de Salud Pública* **2022**, *46*, e53.
12. Araujo, M.; Ossandón, P.; Abarca, A.M.; Menjiba, A.M.; Muñoz, A.M. [Prognosis of patients with COVID-19 admitted to a tertiary center in Chile: A cohort study]. *Medwave* **2020**, *20*, e8066.
13. Lima, E.E.C.; Vilela, E.A.; Peralta, A.; Rocha, M.; Queiroz, B.L.; Gonzaga, M.R.; Piscoya-Díaz, M.; Martinez-Folgar, K.; García-Guerrero, V.M.; Freire, F.H.M.A. Investigating Regional Excess Mortality during 2020 COVID-19 Pandemic in Selected Latin American Countries. *Genus* **2021**, *77*, 1–20.
14. Macchia, A.; Ferrante, D.; Battistella, G.; Mariani, J.; González Bernaldo de Quirós, F. COVID-19 among the Inhabitants of the Slums in the City of Buenos Aires: A Population-Based Study. *BMJ Open* **2021**, *11*, e044592.
15. Machado-Alba, J.E.; Valladales-Restrepo, L.F.; Machado-Duque, M.E.; Gaviria-Mendoza, A.; Sánchez-Ramírez, N.; Usma-Valencia, A.F.; Rodríguez-Martínez, E.; Rengifo-Franco, E.; Forero-Supelano, V.H.; Gómez-Ramirez, D.M.; et al. Factors Associated with Admission to the Intensive Care Unit and Mortality in Patients with COVID-19, Colombia. *PLOS ONE* **2021**, *16*, e0260169.
16. Arias Ramos, D.; Restrepo Rueda, D.L.; Rios Quintero, E.V.; Olaya Gómez, J.C.; Cortés Bonilla, I. Severe and Critical COVID-19 in a Tertiary Center in Colombia, a Retrospective Cross-Sectional Study. *BMC Infect Dis* **2022**, *22*, 247.
17. Schönfeld, D.; Arias, S.; Bossio, J.C.; Fernández, H.; Gozal, D.; Pérez-Chada, D. Clinical Presentation and Outcomes of the First Patients with COVID-19 in Argentina: Results of 207079 Cases from a National Database. *PLOS ONE* **2021**, *16*, e0246793.
18. COVID-19 Excess Mortality Collaborators Estimating Excess Mortality due to the COVID-19 Pandemic: A Systematic Analysis of COVID-19-Related Mortality, 2020-21. *Lancet* **2022**, *399*, 1513–1536.
19. Javier, R.D.; Daniela, O.M.; Daniela, G.A.; Julián, B.J.; Camila, B.O.; Marieliz, R.C.H.; María Luisa, R.Z.; Loreto, R.W.; Cristian, M.A.; Carlos, I.P.; et al. COVID-19 en adultos en el Hospital de Puerto Montt en la primera etapa de la pandemia. *Rev. méd. Chile* **2022**, 465–472.
20. Msemburi, W.; Karlinsky, A.; Knutson, V.; Aleshin-Guendel, S.; Chatterji, S.; Wakefield, J. The WHO Estimates of Excess Mortality Associated with the COVID-19 Pandemic. *Nature* **2022**, *613*, 130–137.
21. Reyes, L.F.; Bastidas, A.; Narváez, P.O.; Parra-Tanoux, D.; Fuentes, Y.V.; Serrano-Mayorga, C.C.; Ortíz, V.; Caceres, E.L.; Ospina-Tascon, G.; Díaz, A.M.; et al. Clinical Characteristics, Systemic Complications, and in-Hospital Outcomes for Patients with COVID-19 in Latin America. LIVEN-Covid-19 Study: A Prospective, Multicenter, Multinational, Cohort Study. *PLOS ONE* **2022**, *17*, e0265529.
22. González, F.J.; Miranda, F.A.; Chávez, S.M.; Gajardo, A.I.; Hernández, A.R.; Guiñez, D.V.; Díaz, G.A.; Sarmiento, N.V.; Ihl, F.E.; Cerda, M.A.; et al. Clinical Characteristics and in-Hospital Mortality of Patients with COVID-19 in Chile: A Prospective Cohort Study. *International Journal of Clinical Practice* **2021**, *75*, e14919.
23. Ñamendys-Silva, S.A.; Alvarado-Ávila, P.E.; Domínguez-Cherit, G.; Rivero-Sigarroa, E.; Sánchez-Hurtado, L.A.; Gutiérrez-Villaseñor, A.; Romero-González, J.P.; Rodríguez-Bautista, H.; García-Briones, A.; Garnica-Camacho, C.E.; et al. Outcomes of Patients with COVID-19 in the Intensive Care Unit in Mexico: A Multicenter Observational Study. *Heart Lung* **2021**, *50*, 28–32.
24. Extended National Consumer Price Index Available online: https://www.ibge.gov.br/en/statistics/economic/prices-and-costs/17129-extended-national-consumer-price-index.html?=&t=series-historicas (accessed on 25 October 2023).
25. INDEC, Instituto Nacional de Estadistica y Censos de la REPUBLICA ARGENTINA INDEC: Instituto Nacional de Estadística Y Censos de La República Argentina Available online: https://www.indec.gob.ar/ (accessed on 30 October 2023).
26. DANE - IPC información técnica Available online: https://www.dane.gov.co/index.php/estadisticas-por-tema/precios-y-costos/indice-de-precios-al-consumidor-ipc/ipc-informacion-tecnica (accessed on 25 October 2023).
27. Chile, I.N.E. Calculadora IPC Available online: https://calculadoraipc.ine.cl/ (accessed on 26 October 2023).
28. Banco Central de Reserva del Perú Available online: https://www.bcrp.gob.pe/ (accessed on 30 October 2023).
29. Giglio, N.D.; Castellano, V.E.; Mizrahi, P.; Micone, P.V. Cost-Effectiveness of Pneumococcal Vaccines for Adults Aged 65 Years and Older in Argentina. *Value Heal. Reg. Issues* **2022**, *28*, S2212–1099.
30. Michelin, L.; Weber, F.M.; Scolari, B.W.; Menezes, B.K.; Gullo, M.C. Mortalidade e custos da pneumonia pneumocócica em adultos: um estudo transversal. *J. bras. pneumol.* **2019**, *45*, e20180374.
31. Andrade, A.L.; Afonso, E.T.; Minamisava, R.; Bierrenbach, A.L.; Cristo, E.B.; Morais-Neto, O.L.; Policena, G.M.; Cmas, D.; Toscano,

C.M. Direct and Indirect Impact of 10-Valent Pneumococcal Conjugate Vaccine Introduction on Pneumonia Hospitalizations and Economic Burden in All Age-Groups in Brazil: A Time-Series Analysis. *PloS one* **2017**, *12*, doi:10.1371/journal.pone.0184204.
32. Biagini, L.; Pezzani, M.; Rojas, R.; Fuentealba, F. Cost-Utility Study of PCV13 Versus PPSV23 in Adults in Chile. *Value in health regional issues* **2018**, *17*, doi:10.1016/j.vhri.2018.09.005.
33. De la Hoz-Restrepo F., C.-O.C.A.-G.N.P.A. Cost-Effectiveness of the Introduction of the Pneumococcal Polysaccharide Vaccine in Elderly Colombian Population. *Vaccine* **2011**, *29*, 7644–7650.
34. Ordóñez, J.E.; Ordóñez, A. A Cost-Effectiveness Analysis of Pneumococcal Conjugate Vaccines in Infants and Herd Protection in Older Adults in Colombia. *Expert Review of Vaccines* **2023**, doi:10.1080/14760584.2023.2184090.
35. Pugh, S.; Wasserman, M.; Moffatt, M.; Marques, S.; Reyes, J.M.; Prieto, V.A.; Reijnders, D.; Rozenbaum, M.H.; Laine, J.; Åhman, H.; et al. Estimating the Impact of Switching from a Lower to Higher Valent Pneumococcal Conjugate Vaccine in Colombia, Finland, and The Netherlands: A Cost-Effectiveness Analysis. *Infectious Diseases and Therapy* **2020**, *9*, 305–324.
36. Urueña, A.; Micone, P.; Magneres, C.; Mould-Quevedo, J.; Giglio, N. Cost-Effectiveness Analysis of Switching from Trivalent to Quadrivalent Seasonal Influenza Vaccine in Argentina. *Vaccines* **2021**, *9*, 335.
37. Tinoco, Y.O.; Azziz-Baumgartner, E.; Rázuri, H.; Kasper, M.R.; Romero, C.; Ortiz, E.; Gomez, J.; Widdowson, M.-A.; Uyeki, T.M.; Gilman, R.H.; et al. A Population-Based Estimate of the Economic Burden of Influenza in Peru, 2009–2010. *Influenza and Other Respiratory Viruses* **2016**, *10*, 301–309.
38. Cost-Effectiveness of Introducing an MF59-Adjuvanted Trivalent Influenza Vaccine for Older Adults in Argentina. *Vaccine* **2020**, *38*, 3682–3689.
39. Cáceres, R.C.; Aj, L.M.; La, P.G.; Ortegón, V.V.; Posso, P.M.; Flórez, E.V.; Gómez, L.E.; Sj, V.F.; Mc, R.L.; Pa, C.L. General Hospitalization and Intensive Care Unit-Related Factors of COVID-19 Patients in Northeastern Colombia: Baseline Characteristics of a Cohort Study. *Cureus* **2023**, *15*, doi:10.7759/cureus.43888.
40. Soto-Cabezas, M.G.; Reyes-Vega, M.F.; Soriano-Moreno, A.N.; Ordoñez-Ibargüen, L.; Martel, K.S.; Flores-Jaime, N.; Chirinos-Saire, J.; Velásquez, J.P.; Munayco, C.V. Comorbilidades Asociadas a La Mortalidad Por COVID-19 En Adultos En Lima, Perú: Un Estudio de Cohorte Retrospectiva. *Revista Peruana de Medicina Experimental y Salud Publica* **2023**, *40*, 132–140.
